# A Type IIb, but Not Type IIa, GnRH Receptor Mediates GnRH-Induced Release of Growth Hormone in the Ricefield Eel

**DOI:** 10.3389/fendo.2018.00721

**Published:** 2018-11-30

**Authors:** Dong Chen, Wei Yang, Shiying Han, Huiyi Yang, Xin Cen, Jiang Liu, Lihong Zhang, Weimin Zhang

**Affiliations:** ^1^Institute of Aquatic Economic Animals and Guangdong Province Key Laboratory for Aquatic Economic Animals, School of Life Sciences, Sun Yat-Sen University, Guangzhou, China; ^2^Guangdong Provincial Engineering Technology Research Center for Healthy Breeding of Important Economic Fish, Sun Yat-Sen University, Guangzhou, China

**Keywords:** ricefield eel *Monopterus albus*, GnRH, GnRHR, somatotrope, Gh release

## Abstract

Multiple gonadotropin-releasing hormone receptors (GnRHRs) are present in vertebrates, but their differential physiological relevances remain to be clarified. In the present study, we identified three GnRH ligands GnRH1 (pjGnRH), GnRH2 (cGnRH-II), and GnRH3 (sGnRH) from the brain, and two GnRH receptors GnRHR1 (GnRHR IIa) and GnRHR2 (GnRHR IIb) from the pituitary of the ricefield eel *Monopterus albus*. GnRH1 and GnRH3 but not GnRH2 immunoreactive neurons were detected in the pre-optic area, hypothalamus, and pituitary, suggesting that GnRH1 and GnRH3 may exert hypophysiotropic roles in ricefield eels. *gnrhr1* mRNA was mainly detected in the pituitary, whereas *gnrhr2* mRNA broadly in tissues of both females and males. In the pituitary, GnRHR1 and GnRHR2 immunoreactive cells were differentially distributed, with GnRHR1 immunoreactive cells mainly in peripheral areas of the adenohypophysis whereas GnRHR2 immunoreactive cells in the multicellular layers of adenohypophysis adjacent to the neurohypophysis. Dual-label fluorescent immunostaining showed that GnRHR2 but not GnRHR1 was localized to somatotropes, and all somatotropes are GnRHR2-positive cells and vice versa at all stages examined. GnRH1 and GnRH3 were shown to stimulate growth hormone (Gh) release from primary culture of pituitary cells, and to decrease Gh contents in the pituitary of ricefield eels 12 h post injection. GnRH1 and GnRH3 stimulated Gh release probably via PLC/IP_3_/PKC and Ca^2+^ pathways. These results, as a whole, suggested that GnRHs may bind to GnRHR2 but not GnRHR1 to trigger Gh release in ricefield eels, and provided novel information on differential roles of multiple GnRH receptors in vertebrates.

## Introduction

Gonadotropin-releasing hormone (GnRH) is a decapeptide best known for its action in releasing gonadotropins through binding to GnRH receptors on gonadotropes of the pituitary in vertebrates. To date, 18 forms of GnRH have been identified in vertebrates and categorized into three classes, GnRH1 (hypophysiotropic GnRHs), GnRH2 (mid-brain chicken GnRH-II), and GnRH3 (salmon GnRH specific to fish) (1). It is well-documented that all vertebrate species possess at least two GnRHs (commonly GnRH2, plus one of either GnRH1 or GnRH3), or all three GnRH types in some fish species (1, 2). In addition to stimulating gonadotropes, GnRHs have also been suggested to regulate other pituitary cells including somatotropes in mammals and fishes (1). In rats, GnRH was shown to increase GH release *in vitro* from hemipituitaries in combination with enkephalin (3) and perifused pituitary cells (4). In teleosts, GnRHs have also been shown to stimulate GH release from cultured pituitary cells of goldfish (5) and tilapia (6), and from pituitary fragments of common carp (7, 8). Moreover, GnRHs have also been demonstrated to upregulate *gh* mRNA in some teleosts including the goldfish (9), common carp (10), blue gourami fish (11), and masu salmon (12).

Multiple types of GnRH receptor (GnRHR) have been reported in mammals, birds, amphibians, and fishes (1, 13–16). Currently, all GnRHRs lacking the C-terminal tail were classified as GnRHR Is, while all others possessing C-terminal tails as GnRHR IIa and GnRHR IIb (17). GnRHR IIa types were further subdivided into IIa-1, IIa-2 and IIa-3 by Williams et al. (18). Humans have lost the functional GnRH receptor GnRHR II and contain only a single functional GnRH receptor GnRHR I, with immunoreactive GnRHR I detected in gonadotropes, thyrotropes, and somatotropes (19). In some other mammals like monkeys, musk shrews, and pigs, both GnRHR I (GnRHR1) and GnRHR II (GnRHR2) are present, with GnRHR1 primarily involved in the regulation of gonadotropes in the pituitary, but the roles for GnRHR2 remain elusive (16). In some non-mammalian vertebrates including ray-finned fishes, amphibian, reptile and bird lineages, the GnRHR I gene appears to have been lost while multiple isoforms of one GnRHR II subtype are often found (1). The pituitary glands of many teleosts, such as the goldfish (20), African catfish (21), medaka (22), spotted green pufferfish (23), European sea bass (24), African cichlid fish *Astatotilapia (Haplochromis) burtoni* (25, 26), Atlantic cod (27), and European eel (28), have been shown to express multiple forms of GnRHRs. However, the information about the types of GnRHRs expressed in somatotropes of teleosts is still very limited with some controversies. Only a minor overlap was observed between mRNA distribution of two forms of GnRHRs (GfA and GfB) with the distribution of somatotropes in the pituitary of goldfish (20). The presence of three forms of GnRHRs, GnRHR1 (belonging to GnRHR IIa group), GnRHR2 (belonging to GnRHR IIb group), and GnRHR3 (belonging to GnRHR IIa group) was demonstrated in somatotropes of tilapia *Oreochromis niloticus* (2). In contrast, in an African cichlid fish (*Haplochromis burtoni*), a teleost very closely related to *Oreochromis niloticus*, the location of *in situ* hybridization signals of GnRH-R2^PEY^ (belonging to GnRHR IIb group) but not GnRH-R1^SHS^ (belonging to GnRHR IIa group) mRNA was shown to be correlated with somatotropes (26). Thus, the GnRH receptor type(s) mediating the regulation of somatotropes by GnRHs in teleosts needs further study. Moreover, the elucidation of the cellular localization of multiple isoforms of GnRHR II in the pituitary may help to unravel the roles for GnRH receptors in these non-mammalian vertebrates as well as in mammals.

The ricefield eel (*Monopterus albus*) is a protogynous hermaphroditic teleost that changes sex from a female stage, through an intersex stage, to a male stage, and also an economically important freshwater fish cultured in China. Previously, we have generated specific antiserum against ricefield eel Gh and shown the presence of immunoreactive somatotropes in the multicellular layers of adenohypophysis adjacent to the neurohypophysis in the pituitary of ricefield eels (29). We have also partially characterized cDNAs encoding three forms of ricefield eel GnRHs (30). In the present study, we are primarily focused on the involvement of GnRH signals in the regulation of somatotropes in ricefield eels. Sequences and expression of three GnRH forms in ricefield eels were further characterized. Two forms of ricefield eel GnRH receptors, GnRHR1 (GnRHR IIa) and GnRHR2 (GnRHR IIb), were identified, and their specific antisera were generated. GnRHR1 and GnRHR2 immunoreactive cells were differentially distributed in the pituitary, with GnRHR2 but not GnRHR1 immunoreactive cells localized to somatotropes from very early developmental stages when somatotropes just appeared. GnRH1 and GnRH3 stimulated Gh release in ricefield eels probably involving PLC/IP_3_/PKC and Ca^2+^ signal transduction pathways.

## Materials and methods

### Animals, tissues, and chemicals

The female, intersexual and male adult ricefield eels (bodylength 30–45 cm and bodyweight 30–60 g) were acquired from a local dealer in Guangzhou (Guangdong, China). The adult fish were anesthetized by immersing into tricaine methanesulphonate (MS222, 0.5 g/L; Sigma) solution and sacrificed by decapitation, after which tissues including the olfactory bulb, telencephalon, hypothalamus, optic tectum-thalamus, cerebellum, medulla oblongata, pituitary, ovary, testis, muscle, spleen, pancreas, heart, liver, kidney, intestines, blood, eyes, and urinary bladder were dissected out, frozen immediately in liquid nitrogen, and stored at −80°C until total RNA extraction or preparation of tissue homogenates, or directly put in ice-cold DMEM (Gibco, MA, USA) medium (the pituitary) for *in vitro* primary culture of pituitary cells. The brain, pituitary and gonadal tissues for histology and immunohistochemistry were fixed in Bouin's solution for 24 h and stored in 70% ethanol until processing. For examination of tissue distribution patterns of gene expression in adult ricefield eels, four set of tissue samples were obtained for females and males, respectively. Ricefield eel larvae and juveniles were obtained from Dazhong Breeding Co. Ltd. (Sichuan, China) and raised in our laboratory. Larvae were collected at the time of 3 days post-fertilization (dpf), and 0, 3, and 7 days post-hatching (dph), and processed as described in our previous report (29). All procedures and investigations were reviewed and approved by the Center for Laboratory Animals of Sun Yat-Sen University, and were performed in accordance with the guiding principles for the care and use of laboratory animals.

Forskolin (F3917) was purchased from Sigma (St. Louis, MO, USA), Rp-cAMPS (sc-24010) from Santa Cruz (TX, USA), U73122 (S8011) from Selleckchem (TX, USA), Xestospongin C (1280) from Tocris Bioscience (Bristol, UK), and GF109203X (HY-13867), and Nifedipine (HY-B0284) from Medchem Express (Monmouth Junction, NJ, USA). The above reagents were carefully diluted with dimethylsulfoxide (DMSO) according to the respective manufacturer's instructions to prepare stock solutions with 10^3^-fold higher than their respective final concentrations, and stored at −80°C. All stocks were diluted to the desired concentrations with culture medium before use. DMSO (0.1%) vehicle was used for all control cultures.

### Total RNA extraction

TRIzol reagent (Invitrogen, MA, USA) was used to extract total RNA from tissues and primary cultures of pituitary cells of ricefield eels. The brain, pituitary, spleen, pancreas, heart, eyes, blood cells, and primary cultures of pituitary cells were directly homogenized in TRIzol reagent (Invitrogen) using BD tuberculin 1 mL syringes with PrecisionGlide™ needles (25 G × 1 in) (Becton, Dickinson and Company, NJ, USA), while other tissues were ground to powder with liquid nitrogen and then homogenized in TRIzol reagent (Invitrogen) using the syringe. Total RNA extracted was then quantified based on the absorbance at 260 and 280 nm in a UV/Visible spectrophotometer (Amersham Biosciences, Buckinghamshire, England). The integrity of RNA was checked with agarose gel electrophoresis.

### Cloning of cDNAs encoding three GnRH forms

3′ and 5′ RACE were carried out to obtain the cDNAs encoding three molecular forms of GnRH in the ricefield eel brain. For 3′ ends, 5 μg of total RNA extracted from the midbrain-diencephalon (including optic tectum-thalamus and hypothalamus) of female eels with TRIzol reagent (Invitrogen, MA, USA) was reverse-transcribed with the dT-AP primer using the Reverse Transcription System (Promega, WI, USA). For the first-round PCR, 4 μL of the first-strand reaction were amplified for *gnrh1, gnrh2*, and *gnrh3* with degenerate forward primers gnrh1-F1, gnrh2-F1, and gnrh3-F, respectively, in combination with AP. PCR was performed in 50 μL final volume containing 5.0 μL 10 × *Ex Taq* Buffer, 2.5 mM MgCl_2_, 0.2 mM dNTP, 0.4 μM of each primer, and 1.25 U *TaKaRa Ex Taq* DNA Polymerase (TaKaRa, DaLian, China). The conditions for PCR were 35 cycles of 0.5 min at 94°C, 1 min at 50°C, and 1 min at 72°C, followed by a final extension for 7 min at 72°C. For the second-round PCR, 1 μL of the first PCR product was amplified with degenerate forward primers gnrh1-F2, gnrh2-F2, and gnrh3-F respectively, in combination with AP. The PCR reaction was identical to the first-round PCR except the annealing temperature was elevated to 53°C. The primers gnrh1-F1, gnrh2-F1, and gnrh3-F were synthesized based on the amino acid sequences of the respective decapeptides, and primers gnrh1-F2 and gnrh2-F2 were synthesized based on the C-terminal amino acid sequences of the GnRH1 and GnRH2 decapeptides, respectively, plus the universal processing site (Gly-Lys-Arg). The second-round PCR products were cloned into pGEM-T Easy Vector (Promega, WI, USA) and sequenced with forward and reverse universal primers using the Bigdye-Terminator kit and an ABI Prism 377 DNA sequencer (Perkin-Elmer, Wellesley, MA, USA).

After determining the nucleotide sequences of the 3′ ends of the three GnRH cDNAs, we used the GeneRacer™ kit (Invitrogen, CA, USA) and the gene-specific primers to identify the 5′ ends of *gnrh* cDNAs according to the manufacturer's instructions. The RACE-ready cDNAs (1 μL) were amplified with gene-specific primers gnrh1-R, gnrh2-R and gnrh3-R for *gnrh1, gnrh2*, and *gnrh3* respectively, in combination with GR5P. The PCR reaction was identical to that described above except the annealing temperature was elevated to 55°C. Primers of *gnrh1, gnrh2* and *gnrh3*, were directed against various regions of the GnRH1, GnRH2, and GnRH3 prohormones, respectively. The primers used for cloning of ricefield eel *gnrh1, gnrh2*, and *gnrh3* were listed in Table S1. The PCR products were cloned into the pGEM-T Easy Vector and sequenced as described above. The full-length cDNA sequences for the three GnRH forms were obtained by combining the 3′- and 5′-end sequences. An additional confirmation was performed by the amplification and sequencing of cDNA fragments containing full-length open reading frames (data not shown). The sequences of GnRHs were analyzed with the methods as described in the Supplementary Material.

Based on deduced amino acid sequences, ricefield eel GnRH1 (pejerrey GnRH, QHWSFGLSPG), GnRH2 (chicken-II GnRH, QHWSHGWYPG), and GnRH3 (salmon GnRH, QHWSYGWLPG) were synthesized by Bachem AG (Bubendorf, Switzerland). The purities of the synthesized peptides are higher than 95% as analyzed by HPLC and their structures were verified by mass spectrometry.

### Cloning of cDNAs encoding two GnRH receptors

Total RNA (1 μg) was extracted from the ricefield eel pituitary glands using TRIzol (Invitrogen, MA, USA) and reversely transcribed with a RevertAid First Strand cDNA Synthesis Kit (Thermo Scientific, MA, USA) according to the manufacturer's instructions. Then 1 μL of the pituitary cDNA was amplified by nested PCR, using the primer set gnrhr-F/R1 for the first round of amplification, and primer set gnrhr-F/R2 for the second round of amplification. PCR was performed in 25 μL final volume containing 2.5 μL 10 × *Ex Taq* Buffer, 2.5 mM MgCl_2_, 0.2 mM dNTP, 0.4 μM of each primer, and 1.25 U *TaKaRa Ex Taq* DNA Polymerase (TaKaRa, DaLian, China). The conditions for PCR were 38 cycles of 0.5 min at 94°C, 0.5 min at 50°C, and 1 min at 72°C, followed by a final extension for 7 min at 72°C. The primers gnrhr-F, gnrhr-R1, and gnrhr-R2 were degenerate primers which were targeted to the conserved regions of various teleost GnRHR open-reading frames. The above nested PCR amplification generated two different initial cDNA fragments corresponding to *gnrhr1* and *gnrhr2*.

Based on the initial cDNA sequences, the 3′ ends of *gnrhr1* and *gnrhr2* cDNAs were obtained by the RACE method using the GeneRacer™ kit (Invitrogen, CA, USA) according to the manufacturer's instructions. The primers were gnrhr1-3'race-F1 and GR3P for the first round and gnrhr1-3'race-F2 and GR3NP for the second round of amplification of *gnrhr1*, and gnrhr2-3'race-F1 and GR3P for the first round and gnrhr2-3'race-F2 and GR3NP for the second round of amplification of *gnrhr2*. The PCR reactions and cycling conditions were the same as the above except that annealing temperature was 55°C. The 5′ ends of *gnrhr1* and *gnrhr2* cDNAs were also extended by the RACE method with nested PCR using gene-specific reverse primers and adaptor primers GR5P and GR5NP. The primers were gnrhr1-5'race-R1 and GR5P for the first round and gnrhr1-5'race-R2 and GR5NP for the second round of amplification of *gnrhr1*, and gnrhr2-5'race-R1 and GR5P for the first round and gnrhr2-5'race-R2 and GR5NP for the second round of amplification of *gnrhr2*. The PCR reactions and cycling conditions were the same as obtaining the 3′ ends. All primers used for ricefield eel *gnrhr1* and *gnrhr2* cloning were listed in Table S1. PCR products of expected sizes were isolated, purified, and sub-cloned into the PGEM-T Easy Vector (Promega, Madison, WI, USA) for DNA sequencing. The full-length cDNA sequences for the two GnRH receptors were obtained by combining the 3′- and 5′-end sequences, which were further confirmed by the amplification and sequencing of cDNA fragments containing the full-length open reading frame (data not shown). The sequences of GnRHRs were analyzed with the methods as described in the Supplementary Material.

### Generation of recombinant polypeptides and antisera

The antiserum against ricefield eel GnRHR1 was generated by immunizing a rabbit with the recombinant C-terminal polypeptide of 78 amino acids. The cDNA sequence encoding amino acid residues 336-414 of ricefield eel GnRHR1 (ARS88253.1) was amplified using gene-specific primers gnrhr1-F and gnrhr1-R (Table S2), subcloned into pET15b, and then expressed without any fusion tag in *E. coli* BL21(DE3) upon induction with IPTG. The recombinant GnRHR1 polypeptide (aa 336-414; designated as GnRHR1 antigen) was gel purified from inclusion bodies and used to immunize New Zealand white rabbit as previously reported (31).

The antiserum against ricefield eel GnRHR2 was generated by immunizing BALB/c mice with a synthetic peptide of 13 amino acids conjugated to KLH. The antigen peptide GKLHPATNNQARN corresponding to the C-terminal (aa 364-376) of ricefield eel GnRHR2 (ARS88254.1) was chemically synthesized, purified and conjugated to KLH by GL Biochem Ltd. (Shanghai, China). The KLH-conjugated GnRHR2 antigen peptide was used to immunize BALB/c mice as previously reported (31).

To examine the specificities of the anti-GnRHR1 and anti-GnRHR2 antisera generated in present study, the cDNA sequences encoding GnRHR1 antigen (aa 336-414) and GnRHR2 polypeptide fragment (aa 295-376) encompassing the synthetic GnRHR2 antigen peptide were PCR amplified with primer sets gnrhr1-F/gnrhr1-R and gnrhr2-F/gnrhr2-R, and cloned into the vector pET32a. These proteins were expressed with TRX fusion tags in *E. coli* BL21(DE3) as above, and designated as GnRHR1-TRX and GnRHR2-TRX, respectively. Moreover, the cDNA fragments encoding GnRHR1 and GnRHR2 mature proteins were PCR amplified with primer sets gnrhr1-pcDNA3.0-F/R and gnrhr2-pcDNA3.0-F/R, respectively, cloned into the pcDNA 3.0 vector. These expression vectors were transfected into COS-7 cells to obtain recombinant mature GnRHR proteins. The primers used in the construction of the expression vectors were also listed in Table S2. The above recombinant proteins were used as negative or positive controls in Western blot and immunohistochemical analysis.

### Western blotting

The recombinant proteins (50 ng) or tissue homogenates (100 μg) were separated by electrophoresis on 12% SDS-PAGE gels and transferred to methanol-activated polyvinylidenefluoride membranes (Merck Millipore, MA, USA). Membranes were blocked with 5% nonfat milk powder at room temperature for 3 h. The blocked membranes were then incubated with the rabbit anti-GnRHR1 antiserum (1:2000 dilution), the mouse anti-GnRHR2 antiserum (1:1000 dilution) or the mouse anti-Actb monoclonal antibody (1:2000 dilution; 60008-1-Ig, ProteinTech Group, Inc., IL, USA) in blocking solution (5% nonfat milk in 0.01 M PBS) at room temperature for 4 h, washed three times with PBST for 10 min, and developed using 1:1000 dilution of the horseradish peroxidase (HRP)-conjugated goat anti-rabbit or anti-mouse IgG (H+L) (Beyotime, Shanghai, China) for 1 h at room temperature. After three 10 min final washes with PBST, the membranes were exposed to a chemiluminescence substrate (BeyoECL Plus kit, P0018, Beyotime) according to the manufacturer's instructions. To confirm the specificity of the anti-GnRHR1 or anti-GnRHR2 antisera, the control membrane was incubated with the primary antiserum pre-adsorbed with an excess of the corresponding full-length GnRHR protein expressed in transfected COS-7 cells.

### Immunohistochemistry

Serial sections (3–4 μm thick) were cut from Bouin's-fixed, paraffin-embedded ricefield eel pituitary glands (together with the brain) and mounted on glass slides coated with poly-L-lysine. The immunohistochemical staining for GnRH and GnRH receptors in the pituitary of ricefield eels were performed essentially as described in our previous work (29). The primary antisera were the rabbit polyclonal antibody AS-691 (1:7,000 dilution; a generous gift from Dr. Masafumi Amano, Kitasato University, Japan) for GnRH1, the rabbit polyclonal antibody 675 (1:2,000 dilution; generously provided by Dr. Judy King, University of Cape Town, South Africa) for GnRH2, and the mouse monoclonal antibody LRH13 (1:2,000 dilution; HACMM02-MSM84; generously provided by Dr. K. Wakabashashi, Gunma University, Japan) for GnRH3, the rabbit polyclonal anti-GnRHR1 antiserum (1:500 dilution) for GnRHR1, and the mouse polyclonal anti-GnRHR2 antiserum (1:500 dilution) for GnRHR2. The rabbit polyclonal antibody AS-691 was generated against sbGnRH (QHWSYGLSPG) conjugated to KLH (32, with personal communication), and cross-reacted with pjGnRH by 40%, with sbGnRH by 100%, and with mGnRH, sGnRH, and cGnRH-II below 0.01 (33). The rabbit polyclonal antibody 675 was generated against cGnRH-II (QHWSHGWYPG) conjugated to BSA, and cross-reacted with cGnRH-II by 100%, and with all other variants of GnRH by < 0.01% (34). The mouse monoclonal antibody LRH13 was generated against the synthetic peptide EHWSYGLRPG (C-terminal free mammalian GnRH) conjugated to bovine thyroglobulin, and recognized the region around Ser^4^-Tyr^5^, which is a common amino acid sequence of GnRH in a variety of animal species (35). LRH13 cross-reacted with sGnRH by 830%, with cGnRH-I by 600%, and with cGnRH-II by 30% (35, 36). In the present study, LRH13 was pre-treated with the supernatant fluid of 25% ricefield eel liver homogenates in PBST. The incubation of sections with primary antisera were done at 4°C and lasted for 40 h in GnRH immunostaining or 12 h in GnRHR immunostaining. Then slides were applied to the secondary antibody, HRP-conjugated goat anti-rabbit or anti-mouse IgG (H+L) (1:1,000 dilution; Beyotime), and finally visualized with 3,3'-diaminobenzidine (DAB) solution, mounted, and digitally photographed with a microscope (Eclipse Ni-U, Nikon, Japan). The specificities of antisera against GnRHs were tested by the corresponding providers, and also validated in other studies (33, 34, 36). To further confirm the specificity of the immunostaining, control sections were incubated with the primary antiserum (in its working solution) pre-adsorbed with an excess of corresponding synthetic GnRHs or recombinant GnRHRs. Additional negative controls included substitution of the primary antiserum with pre-immune serum or PBST and the omission of the secondary antibody.

### Fluorescent immunohistochemistry

The deparaffinized sections were blocked in normal goat serum for 30 min and then incubated in a primary antiserum mixture of the rabbit anti-GnRHR1 (1:500 dilution) with the mouse anti-Gh (1:500 dilution) (29), or the mouse anti-GnRHR2 (1:500 dilution) with the rabbit anti-Gh (1:1,000 dilution) (29) at 4°C overnight. After rinsing with PBST for 10 min three times, the sections were exposed to the secondary antibody, a mixture of Cy3-labeled goat anti-rabbit IgG (H+L) (1:500 dilution; Beyotime) and Alexa Flour 488-labeled goat anti-mouse IgG (H+L) (1:500 dilution; Beyotime), or Cy3-labeled goat anti-mouse IgG (H+L) (1:500 dilution; Beyotime) and Alexa Flour 488-labeled goat anti-rabbit IgG (H+L) (1:500 dilution; Beyotime) for 1 h. After washing three times in PBST for 10 min, the sections were counterstained with DAPI (5 μg/mL; Beyotime) for 15 min and finally coverslipped using an antifade polyvinylpyrrolidone mounting medium (Beyotime). Fluorescent signals for the co-localization of GnRHR2 with Gh were captured using Zeiss LSM 7 *DUO* laser scanning confocal microscope (Germany), and the photographs were overlapped with the Carl Zeiss Application Suite software (ZEN 2011 black edition). Fluorescent signals for the co-localization of GnRHR1 with Gh were captured with a Nikon Eclipse Ni-U microscope (Japan), and the images were overlapped with the NIS-Elements imaging software. The mouse or rabbit anti-Gh antiserum was raised against the recombinant mature polypeptide (aa 18-204) of ricefield eel Gh, which was shown to recognize recombinant Gh but not the other recombinant pituitary hormonal polypeptides including Prl, Sl, Fshb, Lhb, Tshb, and Cga of the ricefield eel (29).

### *In vitro* treatment of primary culture of ricefield eel pituitary cells with GnRHs

The pituitary glands of about 250 ricefield eels (bodylength 30–45 cm and bodyweight 45–60 g) at the intersexual stage were removed and chopped into small pieces. After digesting by trypsin (65 mg/mL; Gibco) at room temperature for 12 min, the dispersed pituitary cells were placed evenly into 24-well plate (Nunc, Denmark) at approximately 1 × 10^6^ cells/mL each well with DMEM (Gibco) containing 10% FBS (Gibco) and cultured at 28°C under an atmosphere of 5% CO_2_. After pre-incubation for 24 h, the medium was changed, and the cells were starved in DMEM without FBS for 12 h. Then cells were washed with DMEM, allowed to rest for 1 h, and subsequently treated with ricefield eel GnRH1 or GnRH3, either in the presence or absence of the PKA inhibitor Rp-cAMPS (50 μM), the PLC inhibitor U73122 (10 μM), the PKC inhibitor GF109203X (20 μM), the IP_3_R inhibitor Xestospongin C (1 μM), or the VSCC blocker nifedipine (10 μM) for the duration as indicated. The doses of the inhibitors used in the present study were chosen as previously reported (37, 38). GnRH stocks (1 mM) were prepared with PBS and diluted to desired final concentrations with DMEM containing 0.1% DMSO before use. Four replicates were performed for each treatment. DMSO (0.1%) vehicle was used for all control cultures. At the end of treatment, culture medium was harvested for measurement of Gh release, and the remaining cells in each well were homogenized individually in RIPA lysis buffer (Beyotime, Shanghai, China) for measurement of cell content of Gh by using a competitive enzyme-linked immunosorbent assay (ELISA), which was established and validated in our laboratory to assay Gh contents in pituitary homogenates and culture medium of pituitary cells of ricefield eels (39). Total production of Gh in individual wells was deduced pro rata based on the protein data for Gh release and cell content. In parallel experiments, pituitary cells were collected and total RNA was extracted for subsequent real-time quantitative PCR analysis of *gh* mRNA levels. In addition, the cAMP production after treatment with GnRH1 or GnRH3 in primary pituitary cells of ricefield eels was also assayed with with a Monoclonal Anti-cAMP Antibody Based Direct cAMP ELISA Kit (catalog number 80203, NewEast Biosciences, Inc., PA, USA), and detailed information was provided in the Supplementary Material. The experiments were repeated three or four times, and similar results were obtained.

### *In vivo* treatment of ricefield eels with GnRHs

Ricefield eels (bodylength 30–35 cm and bodyweight 30–45 g, with mixture of males and females) were purchased from a local dealer in Guangzhou, Guangdong, China, and kept in 50-L plastic tanks in laboratory under a natural photoperiod and room temperature in August 2018, with 16 or 17 fish each tank as a treatment group. The tank water was replaced on alternate days. After acclimatization for 3 days, the ricefield eels were anesthetized with tricaine methanesulphonate (MS222, 0.5 g/L), and received intraperitoneal injections of either GnRH1 or GnRH3 or 0.65% NaCl (vehicle control, 16 or 17 fish per treatment in a tank) GnRH1 or GnRH3 was administered at doses of 0.01 and 0.1 μg/g body weight. The pituitary glands of ricefield eels were dissected out at 12 h after injection and homogenized individually in 200 μL of RIPA lysis buffer (Beyotime) for measurement of cell content of Gh with the competitive ELISA method (39). The gonads were also collected and histologically examined for the sex of individual experimental fish. The data from female and male fish were analyzed separately. The *in vivo* experiments were repeated twice, and similar results were obtained.

### Quantitative real-time PCR analysis

Quantitative real-time PCR (qPCR) was employed to quantify gene expression levels in primary culture of pituitary cells or in tissues of ricefield eels. Total RNA samples isolated from tissues (1 μg) or primary culture of pituitary cells (500 ng) were first treated with RNase-free DNase I (Thermo Scientific, MA, USA) and reverse transcribed with random hexamer primers by using the RevertAid First Strand cDNA Synthesis Kit (Thermo Scientific, MA, USA). Then 1 μL of cDNA template was used for qPCR analysis of *gnrhr1* and *gnrhr2* mRNA levels in tissues and *gh* mRNA levels in primary culture of pituitary cells. The primers were gnrhr1-qF and gnrhr1-qR for *gnrhr1* (KX524496.1), gnrhr2-qF and gnrhr2-qR for *gnrhr2* (KX524497.1), gh-qF and gh-qR for *gh* (AY265351.1), actb-qF and actb-qR for *actb* (AY647143.1), gapdh-qF and gapdh-qR for *gapdh* (FJ873738.1), and hprt1-qF and hprt1-qR for *hprt1* (DQ218476.1). The three housekeeping genes, namely *actb, gapdh*, and *hprt1*, were employed as reference genes for qPCR analysis by following the suggestions of a previous report (40), which is intended to minimize misestimating mRNA expression levels through qPCR due to the potential variations in the expression levels of any single reference gene. The geometric mean expression levels of the three reference genes were used to normalize the expression levels of the target genes. The primers gh-qF and gh-qR for *gh* are located at exon–exon junctions, and upstream and downstream primers for *gnrhr1, gnrhr2, actb, gapdh*, and *hprt1* were targeted to different exons, respectively. The nucleotide sequences of these primers were listed in Table S2. The qPCR analysis was performed in a 10 μL reaction volume containing 0.3 μM of each primer, 1 μL of cDNA template, and 5 μL of Bestar® SybrGreen qPCR mastermix (DBI® Bioscience, Germany), using a Roche LightCycler 480 detection system. The cycling conditions were 5 min at 95°C; 40 cycles of 10 s at 95°C, 15 s at 58°C, and 20 s at 72°C. Data were analyzed by the LightCycler 480 software. The specificity of qPCR amplification was confirmed by melt-curve analysis, gel electrophoresis, and sequencing of PCR products. All samples were run in duplicates and minus reverse transcriptase and no template controls were included in each assay.

The quantification of the mRNA expression levels was performed using a standard curve with tenfold serial dilutions of plasmid containing the corresponding DNA fragment which ranges from 10^1^ to 10^8^ copies, and the correlation coefficients and PCR efficiencies were not < 0.95 and 85%, respectively. The copy numbers of *gnrhr1, gnrhr2, gh*, and the three reference genes were calculated by the LightCycler 480 software based on the corresponding standard curves. The mRNA expression levels of *gnrhr1, gnrhr2*, and *gh* were presented as the copy number ratios to the geometric means of the reference genes.

### Statistical analysis

All data were expressed as mean ± SEM. For the *in vitro* study, data were pooled results from three or four separate experiments. Differences among groups were determined by one-way ANOVA followed by Tukey multiple comparison test using the SPSS 19.0 software (SPSS, Inc., IL, USA). Significance was set at *P* < 0.05.

## Results

### Three forms of prepro-GnRHs were identified in the ricefield eel

The full-length cDNAs encoding three GnRH forms in ricefield eels, namely pjGnRH (pejerrey form, AY858056), cGnRH-II (chicken-II form, AY858054), and sGnRH (salmon form, AY858055) were obtained. All three GnRH precursors possessed the molecular architectures similar to those observed in other GnRH precursors, namely a signal peptide, a GnRH decapeptide, a Gly-Lys-Arg processing site, and a GnRH-associated peptide (GAP) (Figure S1). Ricefield eel pjGnRH, cGnRH-II, and sGnRH were categorized into the branch of GnRH1, GnRH2, and GnRH3 by the phylogenetic analysis (Figure S2) and thus renamed as ricefield eel GnRH1, GnRH2, and GnRH3, respectively.

**Figure 1:**
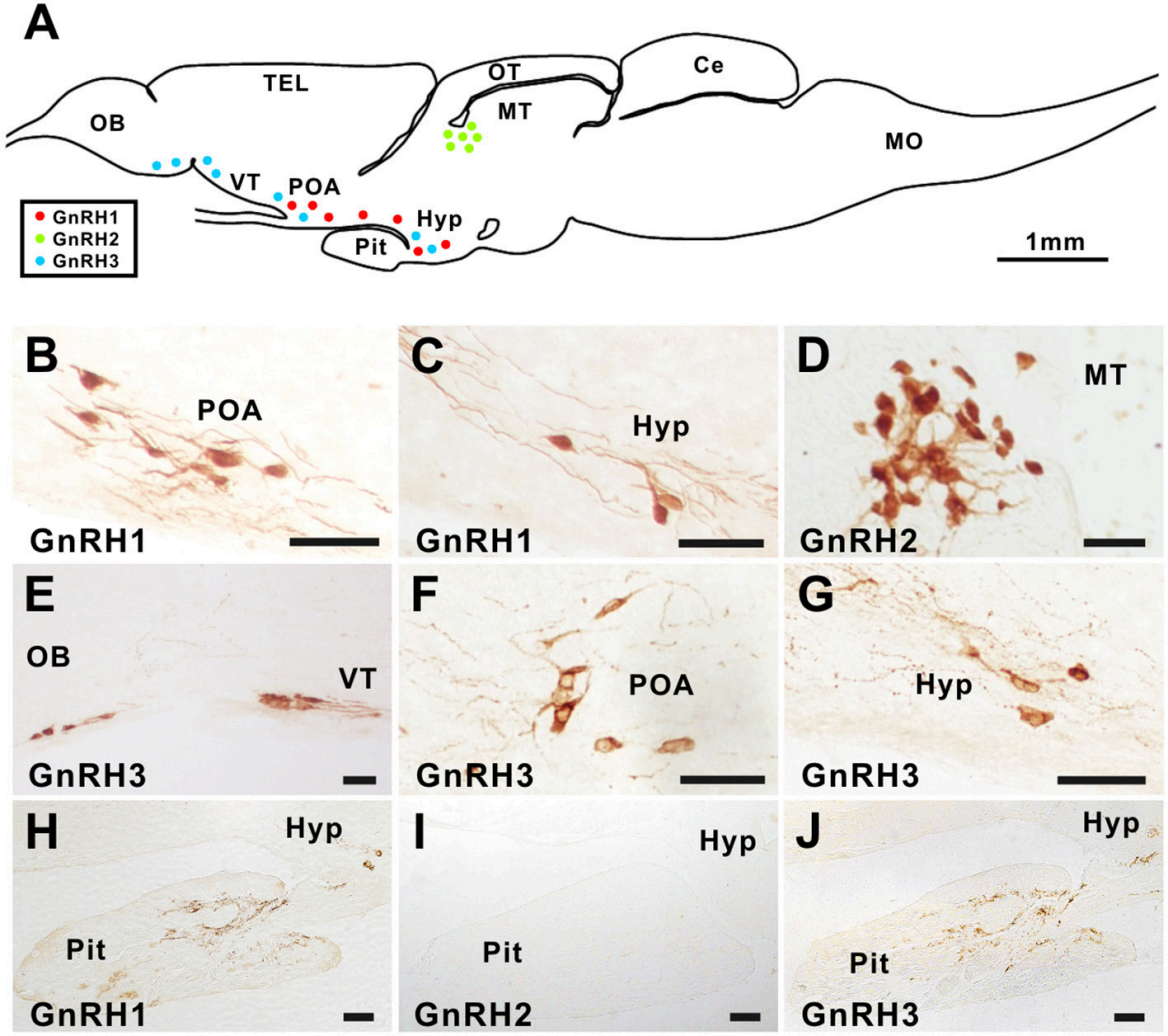
GnRH1, GnRH2, and GnRH3 immunostaining in the brain and pituitary of ricefield eels. Sagittal sections of the brains together with the pituitary gland of ricefield eels were immunoreacted with primary antibodies, the rabbit polyclonal antibody AS-691 for GnRH1 [1:7,000 dilution; **(B,C,H)**], the rabbit polyclonal antibody 675 for GnRH2 [1:2,000 dilution; **(D,I)**], or the mouse monoclonal antibody LRH13 for GnRH3 [1:2,000 dilution; **(E–G,J)**]. After incubation with primary antibodies for 40 h at 4°C, sections were then exposed to the secondary antibody, HRP-conjugated goat anti-rabbit or anti-mouse IgG (H+L) (1:1,000 dilution; Beyotime, Shanghai, China), and finally visualized with 3,3'-diaminobenzidine (DAB) solution, mounted, and digitally photographed with a Nikon Eclipse Ni-U microscope (Japan). **(A)**, the schematic diagram of the ricefield eel brain and pituitary. Red, green, and blue dots in **(A)** represent GnRH1, GnRH2, and GnRH3 immunoreactive neurons, respectively. OB, olfactory bulbs; TEL, telencephalon; VT, ventral telencephalon; POA, preoptic area; OT, optic tectum-thalamus; MT, midbrain tegmentum; Hyp, hypothalamus; Pit, pituitary; Ce, cerebellum; MO, medulla oblongata. Scale bar = 50 μm except where it is specifically designated otherwise.

In the brain of ricefield eels, GnRH1 immunoreactive neurons were observed in the preoptic area and hypothalamus (Figures 1B,C), GnRH2 immunoreactive neurons in the midbrain tegmentum (Figure 1D), and GnRH3 immunoreactive neurons in the olfactory bulb, ventral telencephalon, preoptic area, and hypothalamus (Figures 1E–G). In the pituitary, GnRH1 and GnRH3 but not GnRH2 immunoreactive fibers were detected (Figures 1H–J). GnRH immunoreactive signals disappeared after pre-adsorption of the antiserum with an excess of the corresponding synthetic ricefield eel GnRH peptide, suggesting the specificities of the GnRH immunostaining in the brain and pituitary of ricefield eels (Figure S3).

### A type IIa and a type IIb GnRH receptors were obtained in the ricefield eel

Two GnRH receptors, GnRHR1 (KX524496.1) and GnRHR2 (KX524497.1), were identified from the pituitary of ricefield eels. Ricefield eel *gnrhr1* cDNA encodes a protein of 414 amino acids (Figure S4), and *gnrhr2* cDNA encodes a protein of 376 amino acids (Figure S5). Both receptors contain seven TM domains and N- and C-terminal regions. Phylogenetic analysis (Figure S6) clustered ricefield eel GnRHR1 and GnRHR2 into GnRHR IIa and GnRHR IIb clades, respectively.

### Differential tissue patterns of *gnrhr1* and *gnrhr2* mRNA in the ricefield eel

**Figure 2:**
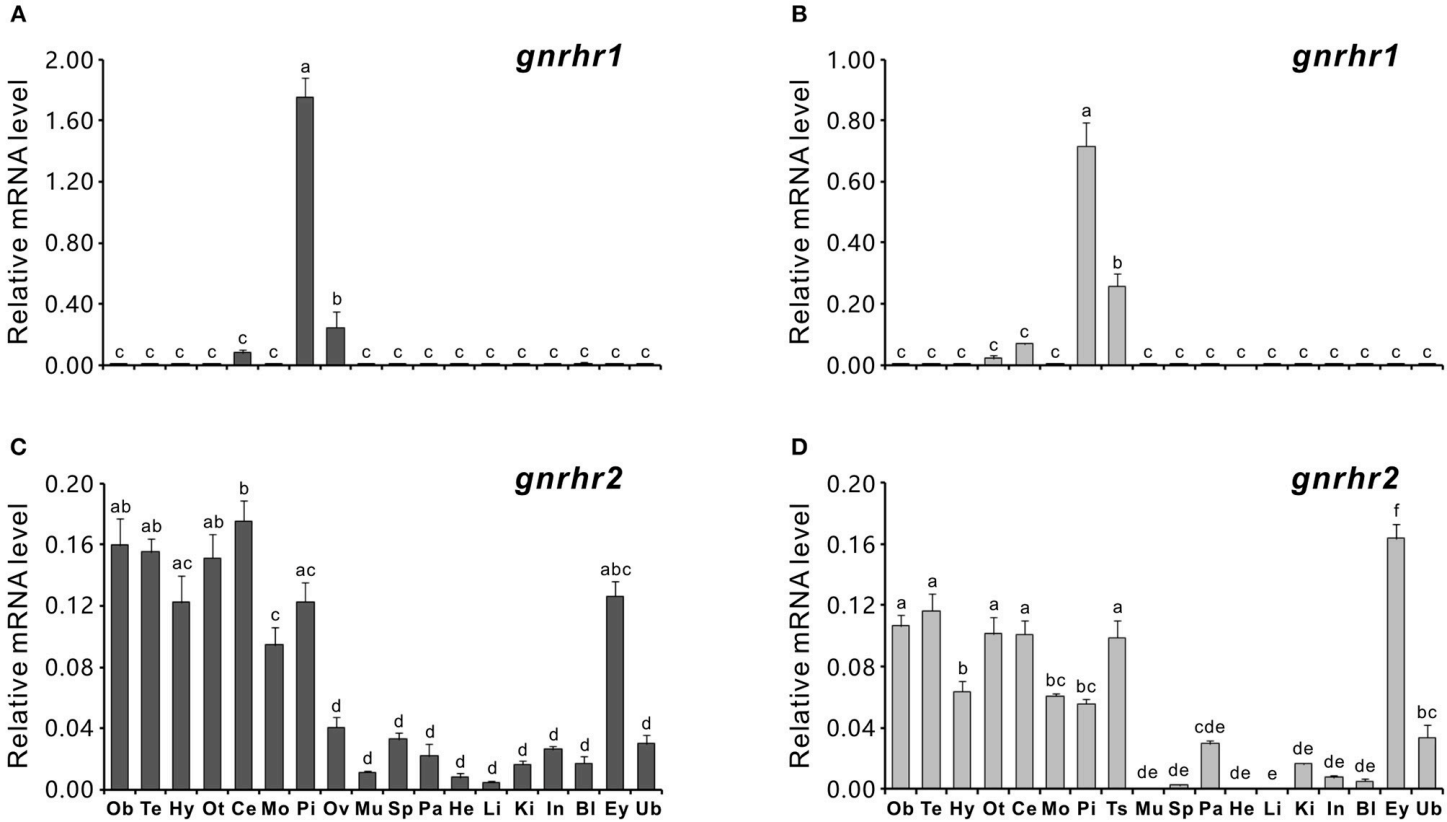
Quantitative real-time PCR analysis of *gnrhr1* and *gnrhr2* mRNA levels in tissues of both female **(A,C)** and male **(B,D)** ricefield eels. Ob, olfactory bulb; Te, telencephalon; Hy, hypothalamus; Ot, optic tectum-thalamus; Ce, cerebellum; Mo, medulla oblongata; Pi, pituitary; Ov, ovary; Ts, testis; Mu, muscle; Sp, spleen; Pa, pancreas; He, heart; Li, liver; Ki, kidney; In, intestines; Bl, blood; Ey, eyes; Ub, urinary bladder. Data represents the mean of normalized expression levels ± SEM of 4 replicates. Means marked with different letters indicate significant differences (*P* < 0.05).

The tissue distribution patterns of *gnrhr1* and *gnrhr2* mRNA were analyzed in both female and male ricefield eels using quantitative real-time PCR. Interestingly, *gnrhr1* mRNA was only detected in restricted tissues, with the highest level in the pituitaries and lower levels in the gonad (ovary or testis) and cerebellum of both female (Figure 2A) and male ricefield eels (Figure 2B). In contrast, *gnrhr2* mRNA was expressed broadly in tissues of both sexes. In the female (Figure 2C), *gnrhr2* mRNA was detected at higher levels in the discrete brain areas (the olfactory bulb, telencephalon, hypothalamus, optic tectum-thalamus, cerebellum, and medulla oblongata), pituitary, and eye, and at lower levels in the ovary, spleen, kidney, intestines, and urinary bladder. In the male (Figure 2D), *gnrhr2* mRNA was detected at higher levels in the discrete brain areas (the olfactory bulb, telencephalon, hypothalamus, optic tectum-thalamus, cerebellum, and medulla oblongata), pituitary, testis, and eye, and at lower levels in the pancreas, kidney, intestine, blood, and urinary bladder, but barely detectable in other tissues examined.

### Specificities of antisera against ricefield eel GnRHR1 and GnRHR2

**Figure 3:**
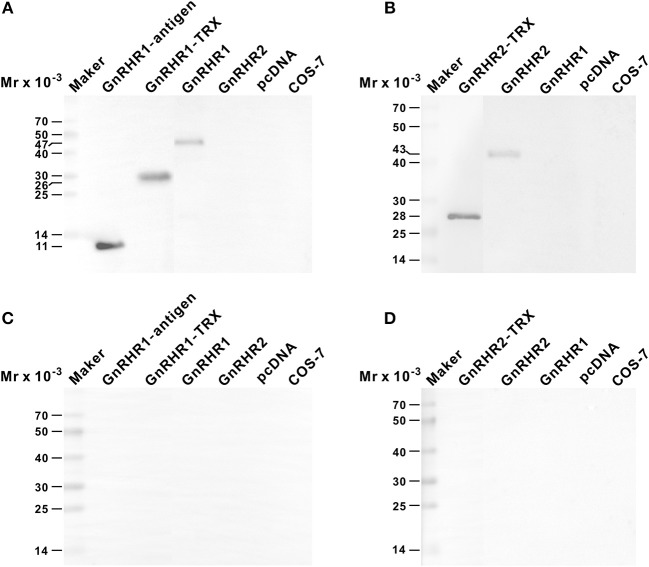
Specificities of anti-GnRHR1 and anti-GnRHR2 antisera against recombinant proteins as determined by Western blot analysis. The recombinant proteins (50 ng) or COS-7 cell extracts (100 μg) were separated on 12% SDS-PAGE gels, processed routinely, and immnoreacted with the rabbit anti-GnRHR1 antiserum [1:2,000 dilution; **(A)**], the mouse anti-GnRHR2 antiserum [1:1,000 dilution; **(B)**], the anti-GnRHR1 antiserum pre-adsorbed with 10 ug/mL of recombinant GnRHR1 expressed in transfected COS-7 cells **(C)**, or the anti-GnRHR2 antiserum pre-adsorbed with 10 ug/mL of recombinant GnRHR2 expressed in transfected COS-7 cells **(D)**. The secondary antibody was 1:1,000 diluted horseradish peroxidase (HRP)-conjugated goat anti-rabbit or anti-mouse IgG (H+L) (Beyotime, Shanghai, China), and the blots were visualized using BeyoECL Plus kit (Beyotime). GnRHR1-antigen, the recombinant GnRHR1 polypeptide used to immunize rabbit; GnRHR1-TRX, the region corresponding to GnRHR1 antigen expressed as thioredoxin (TRX) fusion protein; GnRHR2-TRX, GnRHR2 polypeptide region encompassing the synthetic antigen peptide expressed as TRX fusion protein. GnRHR1 and GnRHR2, the recombinant full-length ricefield eel GnRHR1 and GnRHR2 proteins expressed in transiently transfected COS-7 cells. pcDNA, extracts of COS-7 cells transfected with the empty vector pcDNA3.0. COS-7: extracts of COS-7 cells.

The rabbit antiserum against ricefield eel GnRHR1 could recognize GnRHR1 antigen, GnRHR1 antigen-TRX fusion peptide, and GnRHR1 mature protein expressed in transfected COS-7 cells, but did not cross-react with GnRHR2 (Figure 3A). The mouse antiserum against ricefield eel GnRHR2 could recognize the recombinant C-terminal polypeptide encompassing the synthetic GnRHR2 antigen and GnRHR2 mature protein expressed in transfected COS-7 cells, but did not cross-react with GnRHR1 (Figure 3B). Pre-absorption of the antiserum with corresponding mature GnRHR protein expressed in transfected COS-7 cells abolished all the signals (Figures 3C,D), suggesting that both antisera are of high specificities. Moreover, Western blot analysis of tissue extracts with anti-GnRHR1 or anti-GnRHR2 antiserum revealed specific protein bands of approximately the expected size corresponding to GnRHR1 (47 kDa) or GnRHR2 (43 kDa) from the pituitary, brain, testis and ovary, but not in the liver (Figures S7A,C). When the antiserum was pre-absorbed with an excess of the corresponding GnRHR expressed in transfected COS-7 cells, all the specific bands disappeared (Figures S7B,D), further confirming the specificities of the antisera generated.

### Differential immunolocalization of ricefield eel GnRHR1 and GnRHR2 in the pituitary

**Figure 4:**
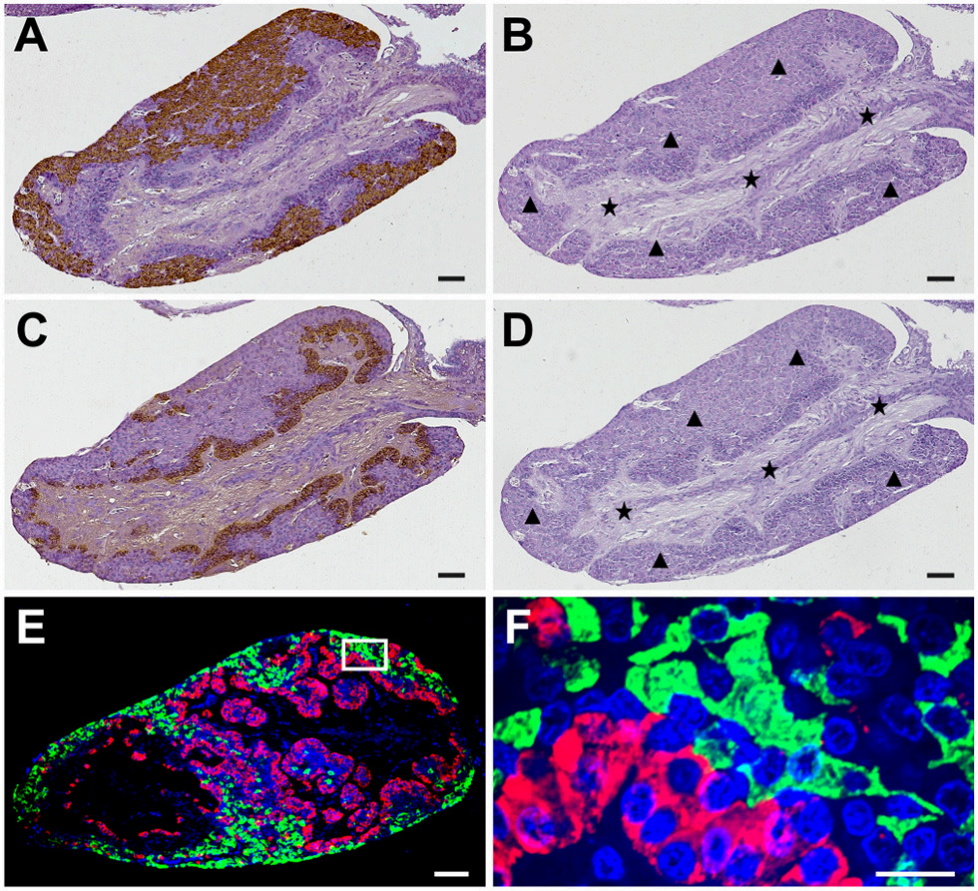
Specificities of GnRHR1 and GnRHR2 immunoreactivities in the pituitary of male ricefield eels as determined by immunohistochemical analysis. Sagittal sections of the pituitary gland were immunoreacted with the rabbit anti-GnRHR1 antiserum [1:500 dilution; **(A)**], the pre-adsorbed anti-GnRHR1 antiserum by 10 ug/mL of recombinant GnRHR1 expressed in transfected COS-7 cells **(B)**, the mouse anti-GnRHR2 antiserum [1:500 dilution; **(C)**], the pre-adsorbed anti-GnRHR2 antiserum by 10 ug/mL of recombinant GnRHR2 expressed in transfected COS-7 cells **(D)**, or the mixture of the rabbit anti-GnRHR1 (1:500 dilution) with the mouse anti-GnRHR2 (1:500 dilution) antisera **(E,F)**. The secondary antibody was 1:1,000 diluted horseradish peroxidase (HRP)-conjugated goat anti-rabbit or anti-mouse IgG (H+L) (Beyotime, Shanghai, China) for **(A–D)**, and the mixture of Alexa Flour 488-labeled goat anti-rabbit IgG (H+L) (1:500 dilution) and Cy3-labeled goat anti-mouse IgG (H+L) (1:500 dilution) for **(E)**. DAPI was used to stain the nuclei blue. Immunostaining (brown) in **(A–D)** was visualized by the DAB chromogen and counterstained with hematoxylin. The black triangles and stars indicate the adenohypophysis and neurohypophysis tissues, respectively. The image of **(E)** was captured and analyzed with a Nikon i-E confocal microscope equipped with a CSU-W1 spinning-disk head (Yokogawa, Tokyo, Japan) for immunofluorescent staining of GnRHR1 (green) and GnRHR2 (red). **(F)** is the higher magnification of the boxed area in **(E)**. Sagittal sections of ricefield eel pituitary glands were shown here with the rostral (anterior) to the left. Scale bar = 50 μm except the image of **(F)** was 10 μm.

Immunostaining of the adjacent pituitary sections showed that GnRHR1-immunoreactive cells were predominantly localized to an extensive area in the peripheral of the adenohypophysis (Figure 4A), whereas GnRHR2-immunoreactive cells were mainly located in the multicellular layers of adenohypophysis adjacent to the neurohypophysis (Figure 4C). Pre-adsorption of the primary antiserum with an excess of corresponding GnRHR expressed in transfected COS-7 cells abolished all positive immunoreactive signals (Figures 4B,D), suggesting the specificities of immunostaining for ricefield eel GnRHR1 and GnRHR2 in the pituitary. Furthermore, fluorescent GnRHR1 and GnRHR2 signals were not overlapped in the pituitary (Figures 4E,F). These results demonstrated differential cellular localization of GnRHR1 and GnRHR2 in the pituitary of ricefield eels.

### GnRHR2 but not GnRHR1 immunostaining co-localizes with Gh in the pituitary

**Figure 5:**
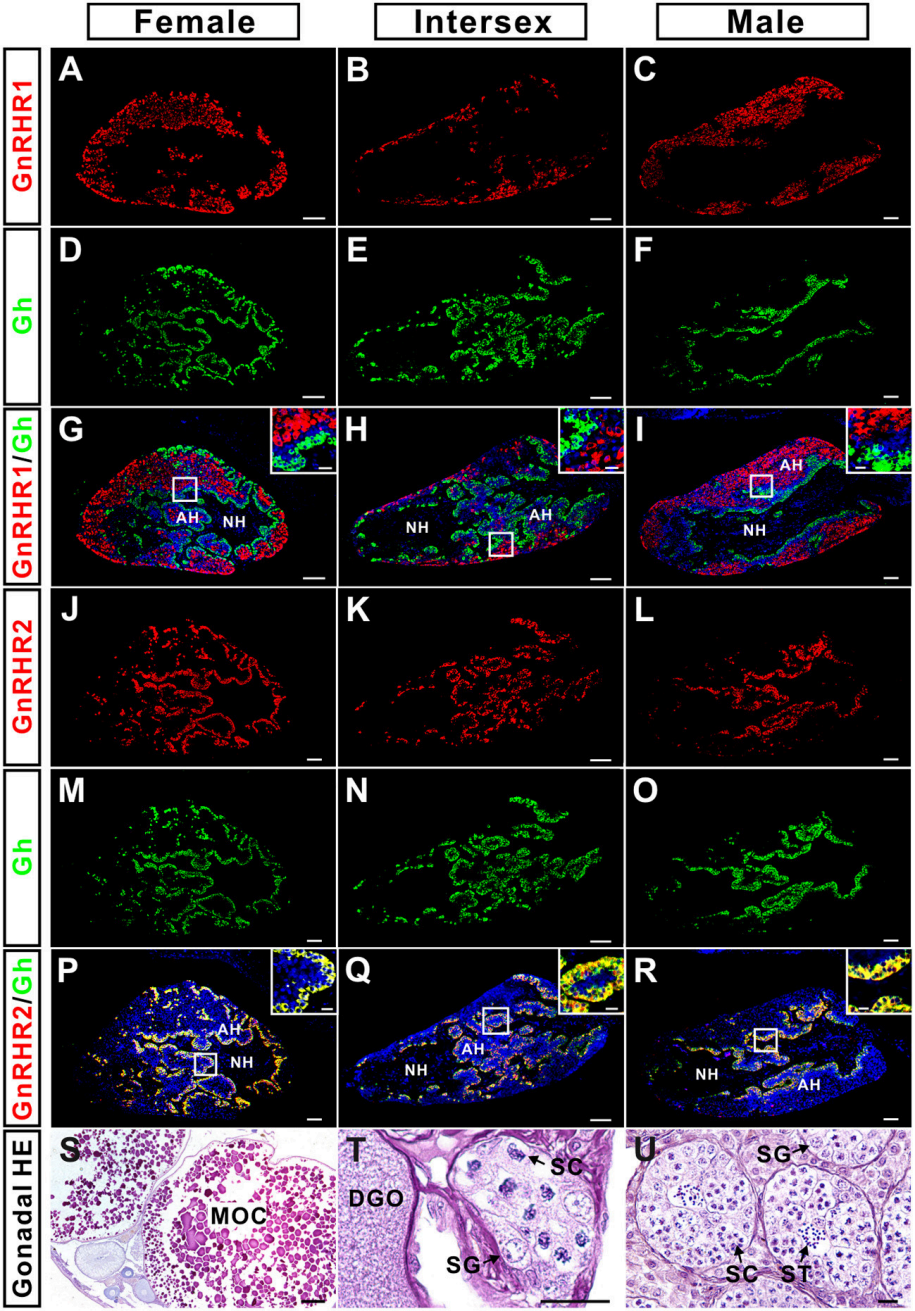
Co-localization of GnRHR1 (red) or GnRHR2 (red) with Gh (green) immunostaining in the pituitary of ricefield eels at female, intersexual, and male stages. The mixture of the rabbit anti-GnRHR1 (1:500 dilution) with the mouse anti-Gh (1:500 dilution), or the mouse anti-GnRHR2 (1:500 dilution) with the rabbit anti-Gh (1:1,000 dilution) were used as the primary antisera. The mixture of Cy3-labeled goat anti-rabbit IgG (H+L) (1:500 dilution) and Alexa Flour 488-labeled goat anti-mouse IgG (H+L) (1:500 dilution), or Cy3-labeled goat anti-mouse IgG (H+L) (1:500 dilution) and Alexa Flour 488-labeled goat anti-rabbit IgG (H+L) (1:500 dilution) were used as the secondary antibodies (Beyotime, Shanghai, China). DAPI was used to stain the nuclei blue, and the neurohypophysis (NH) is mostly devoid of stain. The insets are higher magnification of the boxed areas within each image. The overlapping of the red with the green color generated a yellow color. **(A–C)**, GnRHR1 immunostaining in the pituitary of female, intersexual, and male fish, respectively; **(D–F)**, Gh immunostaining in the pituitary of female, intersexual, and male fish, respectively; **(G–I)**, overlapping of A and D, B and E, and C and F, respectively; **(J–L)**, GnRHR2 immunostaining in the pituitary of female, intersexual, and male fish, respectively; **(M–O)**, Gh immunostaining in the pituitary of female, intersexual, and male fish, respectively; **(P–R)**, overlapping of J and M, K and N, and L and O, respectively. **(S–U)**, HE-stained images of gonadal sections of the experimental fish at female, intersexual, and male stages, respectively. AH, adenohypophysis; MOC, mature oocyte; DGO, degenerating oocyte; SG, spermatogonium; SC, spermatocyte; ST, spermatid. Sagittal sections of ricefield eel pituitaries were shown here with the rostral (anterior) to the left. Scale bar = 50 μm except the insets were 10 μm.

**Figure 6:**
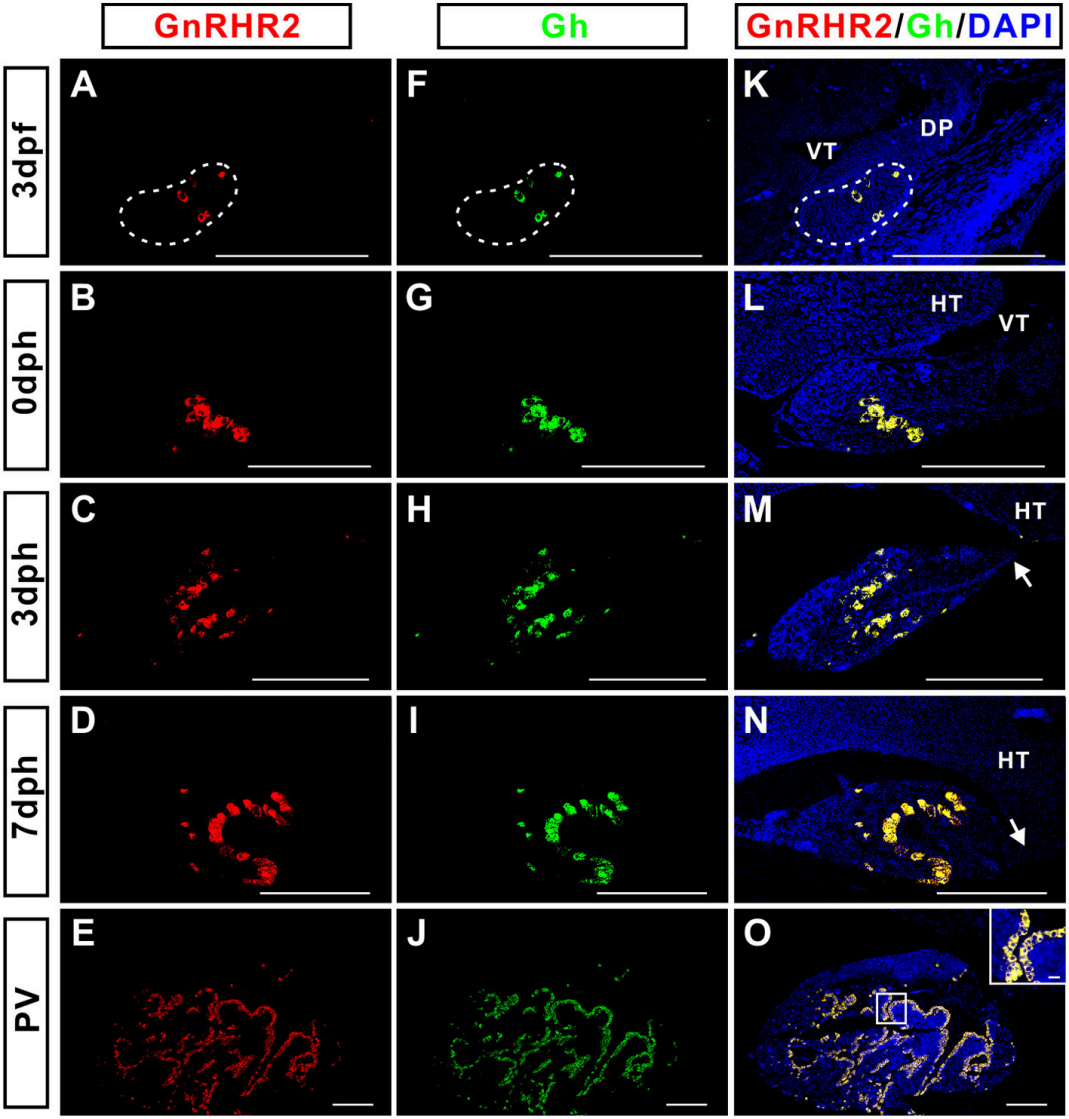
The ontogenic analysis of the co-localization of GnRHR2 (red) with Gh (green) immunostaining in the pituitary of ricefield eels from 3 days post-fertilization (dpf) to pre-vitellogenic stage (PV). The mixture of the mouse anti-GnRHR2 (1:500 dilution) with the rabbit anti-Gh (1:1,000 dilution) were used as the primary antisera. The mixture of Cy3-labeled goat anti-mouse IgG (H+L) (1:500 dilution) and Alexa Flour 488-labeled goat anti-rabbit IgG (H+L) (1:500 dilution) were used as the secondary antibodies (Beyotime, Shanghai, China). DAPI was used to stain the nuclei blue. The inset is a higher magnification of the boxed areas within the image. The overlapping of the red with the green color generated a yellow color. The pituitary placode is delineated with a dashed line at 3 dpf. At 3 and 7 days post-hatching (dph), the pituitary gland became more distinguished in shape, with the formation of the pituitary stalk (*white arrows*). Sagittal sections of ricefield eel pituitaries were shown here with the rostral (anterior) to the left. **(A–E)**, GnRHR2 immunostaining in the pituitary of ricefield eels at 3 dph, 0 dph, 3 dph, 7 dph, and PV stages, respectively; **(F–J)**, Gh immunostaining in the pituitary of ricefield eels at 3 dph, 0 dph, 3 dph, 7 dph, and PV stages, respectively; **(K–O)**, overlapping of A and F, B and G, C and H, D and I, and E and J, respectively. VT, ventricle; DP, diencephalon; HT, hypothalamus. Scale bar = 50 μm except the inset (10 μm).

The distribution of GnRHR2 immunoreactive signals in the pituitary (Figure 4) exhibited a pattern similar to that of Gh reported previously (29). Therefore, the possible co-localization of Gh with GnRHR1 or GnRHR2 was examined by dual fluorescent immunohistochemistry in the pituitary of adult ricefield eels at different sexual stages (Figure 5). GnRHR2 but not GnRHR1 immunostaining was co-localized with Gh in the pituitary of adult ricefield eels at all sexual stages examined (Figure 5). All the GnRHR2 positive cells are somatotropes and vice versa. We also examined the colocalization of GnRHR2 with Gh from larvae to vitellogenic females. The colocalization of GnRHR2 with Gh could be detected as early as 3 dpf (Figure 6), when somatotropes just appeared (29). Along with development, both GnRHR2 and Gh immunoreactive signals were increased and always perfectly co-localized (Figure 6).

### GnRH1 and GnRH3 stimulated Gh release

**Figure 7:**
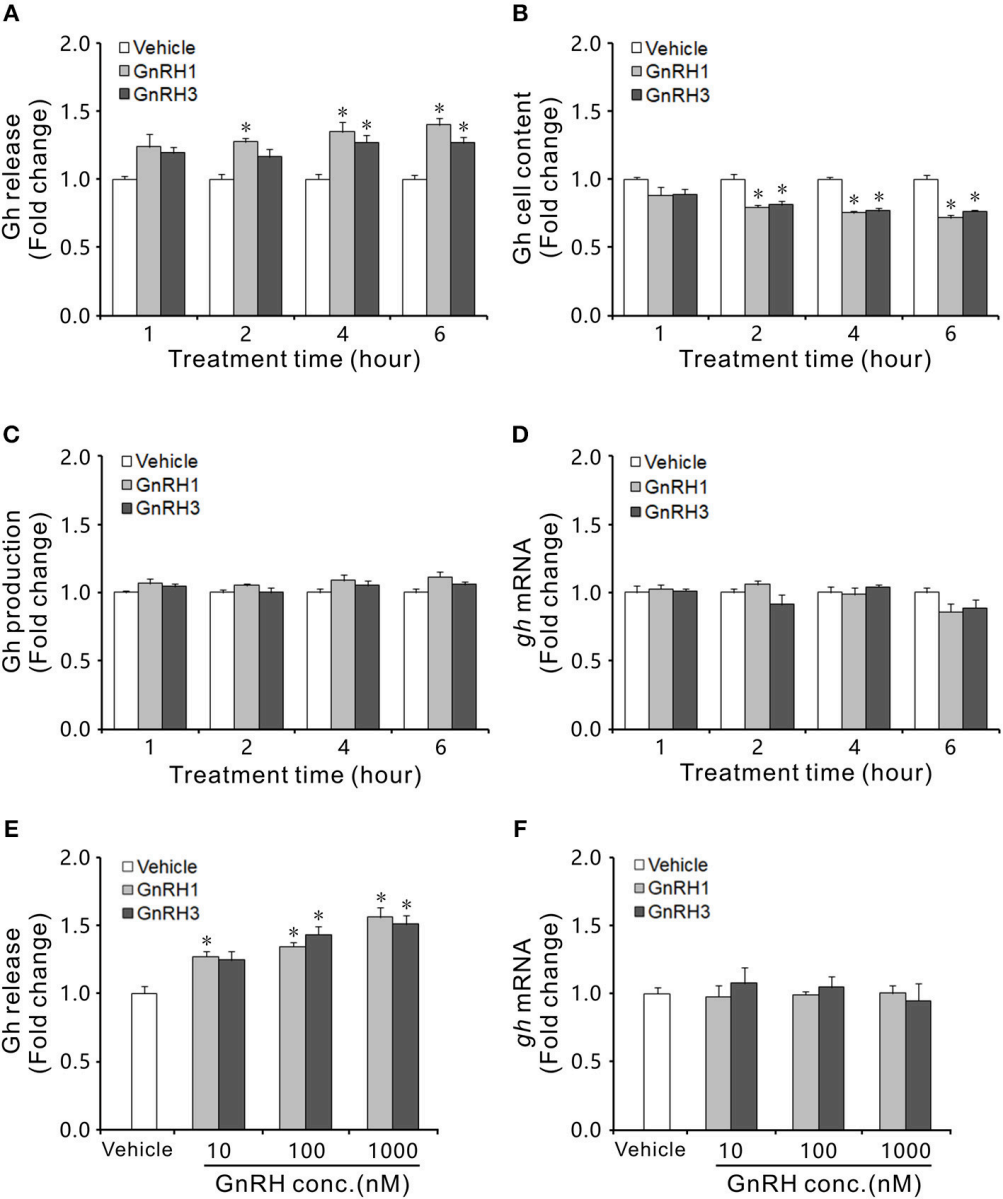
Effects of GnRH1 and GnRH3 on Gh synthesis and release in primary culture of pituitary cells of intersexual ricefield eels. Time course of GnRH1 (100 nM) and GnRH3 (100 nM) treatment on Gh release **(A)**, cell content **(B)**, total production **(C)**, and mRNA expression **(D)** in primary culture of pituitary cells. **(E,F)**, Dose-dependence of 4-h treatment with increasing levels of GnRH (10–1,000 nM) on Gh release and *gh* mRNA, respectively. After drug treatment, culture medium from individual well was harvested for measurement of Gh release by ELISA, and cell lysate was prepared for monitoring Gh content in pituitary cells. In parallel experiments, total RNA was isolated for real-time quantitative PCR of *gh* mRNA expression. Data were expressed as fold change relative to the corresponding controls. Bars represent means ± SEM (*n* = 4). The groups denoted by the asterisks (^*^) represent significant differences (*P* < 0.05) *vs*. the corresponding vehicle controls.

**Figure 8:**
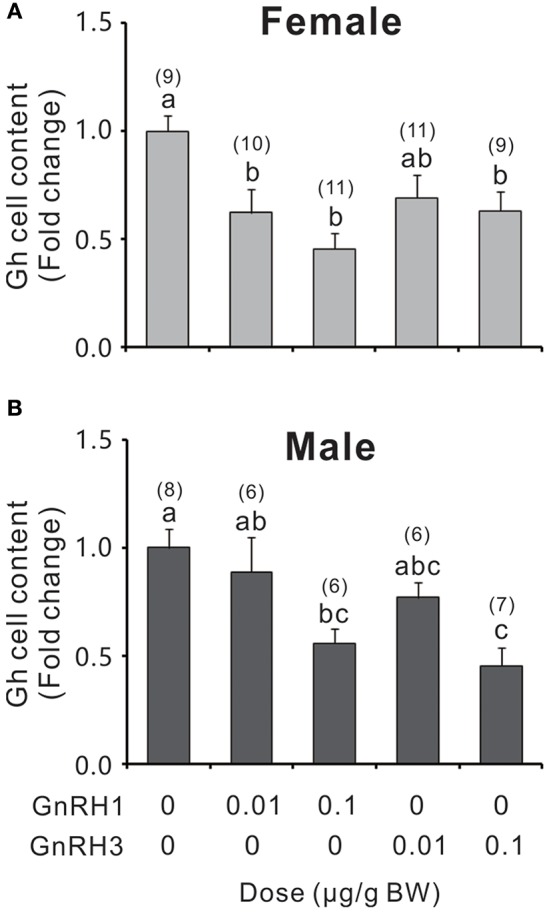
Relative Gh levels in the pituitary of female **(A)** and male **(B)** ricefield eels after intraperitoneal injection of GnRH1 or GnRH3 (0.01 and 0.1 μg/g bodyweight), or 0.65% NaCl (Vehicle control) for 12 h. Numbers in parentheses represent the number of fish for each treatment. BW: bodyweight. Data were expressed as fold change relative to the corresponding vehicle control. Bars represent mean ± SEM (*n* = 6–11). Mean values marked with different letters are significantly different from each other (*P* < 0.05).

GnRH1 and GnRH3 immunoreactive fibers were shown to be in close proximity to Gh cells (Figure S8), even with some co-localizations in the pituitary of ricefield eels (Figures S8B,D), suggesting the potential regulation of Gh cells by GnRHs in ricefield eels. The effects of GnRH1 and GnRH3 on Gh synthesis and release were firstly examined in primary culture of pituitary cells. Time-course experiments showed that the amounts of Gh released in GnRH1 or GnRH3 (100 nM)-treated cells were higher or significantly higher than those of control cells at 2, 4, and 6 h of incubation (Figure 7A). In contrast, the cellular Gh contents of GnRH (100 nM)-treated cells were significantly lower than those of the control cells at 2, 4, and 6 h of incubation (Figure 7B). The total amounts of Gh production (Figure 7C) and *gh* mRNA levels (Figure 7D) were not significantly different between GnRH (100 nM)-treated and control cells. Dose-dependent studies showed that 4-h incubation with increasing levels of GnRH (10 nM-1000 nM) also increased Gh release in a dose-related fashion (Figure 7E), but *gh* transcript levels were not significantly altered (Figure 7F). In addition, *in vivo* treatment with GnRH1 or GnRH3 (0.01 and 0.1 μg/g BW) significantly decreased Gh contents in the pituitary of both females and males 12 h post injection (Figure 8).

### GnRH1 and GnRH3-stimulated Gh release involve PLC/IP_3_/PKC and Ca^2+^ pathways

**Figure 9:**
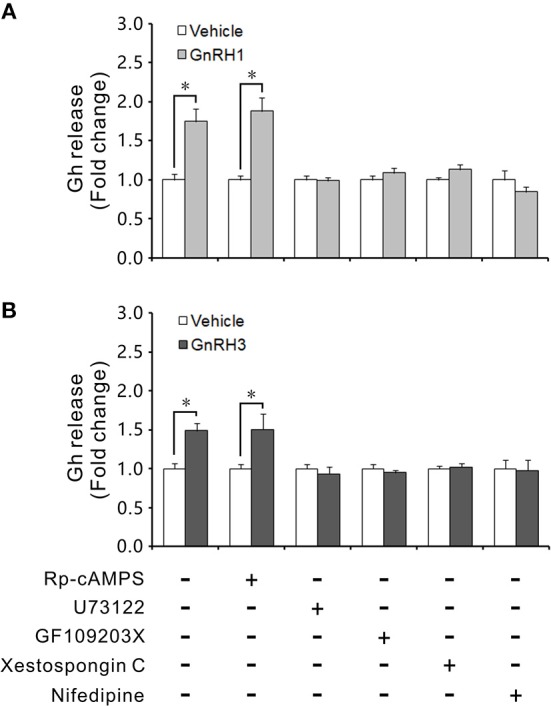
Effects of intracellular signaling pathway inhibitors on GnRH-stimulated Gh release. The primary cultured pituitary cells of intersexual ricefield eels were pre-incubated for 24 h before being treated with 100 nM of GnRH1 **(A)** or GnRH3 **(B)** in the presence or absence of inhibitors Rp-cAMPS (50 μM), U73122 (10 μM), GF109203X (20 μM), Xestospongin C (1 μM), or Nifedipine (10 μM) for 4 h, respectively. After drug treatment, culture medium was harvested for measurement of Gh release by competitive ELISA. Data were expressed as fold change relative to the vehicle control. Bars represent means ± SEM (*n* = 3). ^*^P < 0.05 vs.the vehicle control.

Incubation with GnRH1 or GnRH3 for 4 h could significantly increase Gh release from ricefield eel pituitary cells (Figure 9). Addition of the PKA inhibitor Rp-cAMPS (50 μM) could not block the stimulatory effects of GnRH1/GnRH3 on Gh release (Figure 9). In consistency, GnRH1 or GnRH3 (100 nM) could not stimulate cAMP production in primary culture of pituitary cells (Figure S9). Addition of the PLC inhibitor U73122 (10 μM), the PKC inhibitor GF109203X (20 μM), the IP_3_R inhibitor Xestospongin C (1 μM), or the VSCC blocker nifedipine (10 μM) could abolish the stimulation of GnRH1 or GnRH3 on Gh release (Figure 9). These results suggested that PLC/IP_3_/PKC and Ca^2+^ were involved in mediating GnRH1 and GnRH3-induced Gh release in ricefield eels.

## Discussion

As in most of other teleosts (1), the presence of three GnRH forms, namely GnRH1, GnRH2, and GnRH3 in ricefield eels, was substantiated by the present data, our previous report (30), and a recent publication on the alternative splicing of ricefield eel GnRH2 (41). In consistency with the general expression patterns of GnRH1, GnRH2, and GnRH3 in the brain of vertebrates (1, 42), immunohistochemistry detected ricefield eel GnRH1 and GnRH3 neurons in the preoptic area and hypothalamus, and GnRH2 neurons in the midbrain. Unlike mammals, a median eminence has not been identified in most fish, and hypophyseal axon terminals project to the pituitary to control the secretion of pituitary hormones by direct innervation (43) as well as through the vasculature within the pituitary (44). In any case, the presence of GnRH immunoreactive fibers in the pituitary is suggestive of hypophysiotropic roles of the corresponding GnRH form. The types of GnRH in the pituitary appears to vary among teleost species. In goldfish, both cGnRH-II (GnRH2) and sGnRH (GnRH3) were detected in the pituitary (45, 46). In zebrafish, in addition to the hypophysiotropic GnRH3, cGnRH-II (GnRH2) fibers have also been detected in the pituitary recently (47). In contrast, both GnRH1 and GnRH3 but not GnRH2 fibers were observed in the pituitary of European sea bass (*Dicentrarchus labrax*) (48). Similarly, our present study also detected immunostaining for GnRH1 and GnRH3 but not GnRH2 in the pituitary of the ricefield eels, a species belonging to Perciformes as the European sea bass. These results suggest that both GnRH1 and GnRH3 may have hypophysiotropic roles in the ricefield eel and European sea bass. As the antibody LRH13 has been shown to cross-react with other forms of GnRH besides GnRH3 (36), GnRH3 immunostaining in the brain and pituitary of ricefield eels should be interpreted with caution. In this context, an *in situ* hybridization study with *gnrh3* cDNA probes is needed to confirm the localization of GnRH3 cells in the brain of ricefield eels.

Physiological roles of GnRH are mediated through GnRHRs on target tissues. Multiple GnRHR types have been identified from different species of vertebrates, which was proposed to be categorized into three distinct subgroups: GnRHR I, GnRHR IIa, and GnRHR IIb (17). Our present study cloned two forms of GnRH receptors, designated as GnRHR1 and GnRHR2, from the pituitary of ricefield eels, which were clustered in GnRHR IIa and GnRHR IIb clades, respectively. The expression of ricefield eel GnRHR1 and GnRHR2 in the pituitary were further confirmed by qPCR analysis. Similarly, multiple types of GnRH receptors have been detected in the pituitary of mammals and other teleosts. In addition to GnRHR1 (GnRHR I), GnRHR2 (GnRHR II) is expressed in the pituitary of marmoset (49) and pig (50, 51), respectively. It has also been shown that two forms of GnRH receptors are expressed in the pituitary of goldfish *Carassius auratus* (20), African catfish *Clarias gariepinus* (21), and African cichlid fish *Astatotilapia (Haplochromis) burtoni* (25, 26), and three forms of GnRH receptors in the pituitary of Medaka *Oryzias latipes* (22, 52) and tilapia (2). The spotted green pufferfish *Tetraodon nigroviridis* expresses five isoforms of GnRH receptors, of which three forms (GnRHR1-1, GnRHR1-3, and GnRHR2-1) are expressed in the pituitary (23). The European sea bass *Dicentrarchus labrax* also expresses five isoforms of GnRH receptors, of which four forms are expressed in the pituitary (24). These studies suggest that multiple GnRH receptors may be involved in mediating hypophysiotropic roles of GnRHs in vertebrates.

In agreement with diverse extra-pituitary roles of GnRH like neuromodulation, gonadal regulation (53, 54), and cell proliferation regulation (55, 56), GnRH receptors have been shown to be expressed in a wide range of tissues besides the pituitary gland in ricefield eels and other vertebrates (23, 26, 57–59). Notably, differential mRNA tissue distribution patterns were detected for ricefield eel *gnrhr1* and *gnrhr2*, with *gnrhr1* expressed in very restricted tissues including the cerebellum, pituitary, and gonads, whereas *gnrhr2* expressed widely in various tissues, including the discrete brain areas, pituitary, gonads, spleen, kidney, intestines, eyes, urinary bladder, and pancreas. Similarly, mammalian GnRHR2 genes were also ubiquitously expressed in tissues including the pituitary (16). In African cichlid fish *Haplochromis burtoni*, GnRH-R (belonging to GnRHR IIb group) mRNA has also been shown to be widely distributed in the brain, pituitary, retina, testis, kidney, and muscle (60). The differential expression profiles of GnRH receptors in ricefield eels suggest that both GnRHR1 and GnRHR2 may mediate GnRH actions in the pituitary and gonads, whereas GnRHR2 may play dominant roles in mediating GnRH actions in the brain and other peripheral tissues.

The presence of GnRH receptors on somatotropes was firstly reported in goldfish using biotinylated GnRH analogs (61), then Illing and collaborators (20) demonstrated a minor overlap between distribution of GnRH receptors and distribution of somatotropes using *in situ* hybridization. In male tilapia *Oreochromis niloticus*, all three forms of GnRHRs have been detected in the laser-capture microdissected somatotropes (2). Interestingly, our present study showed that immunostaining for ricefield eel GnRHR1 and GnRHR2 as revealed with homologous antisera was differentially distributed, with GnRHR2 but not GnRHR1 localized to somatotropes in the pituitary of ricefield eels. In the African cichlid fish (*Haplochromis burtoni*), the mRNA expression of GnRH-R2^PEY^ (belonging to GnRHR IIb group) but not GnRH-R1^SHS^ (belonging to GnRHR IIa group) was shown to be correlated with somatotropes, suggesting that somatotropes may express GnRH-R2^PEY^ but not GnRH-R1^SHS^ (26). Considering that both GnRH-R2^PEY^ and ricefield eel GnRHR2 belong to the GnRHR IIb group, and GnRH-R1^SHS^ and ricefield eel GnRHR1 belong to the GnRHR IIa group, there seems a certain conservation in sequences and functions of GnRH receptor types regulating somatotropes in some teleosts. In the pituitary of ricefield eels at all sexual stages examined, the GnRHR2-positive cells are somatotropes and vice versa. The presence of GnRHR2 in somatotropes of ricefield eels could be traced back to larvae stages as early as 3 dpf (days post-fertilization), when somatotropes just appear (29). In tilapia, similarly, GnRH receptors have also been detected in somatotropes of females, immature males, and mature males (2, 62), and GnRH receptors could be observed in somatotropes on day 8 after fertilization when the pituitary anlage was first recognized (62). In the African catfish, however, somatotrophs were not found to carry GnRH receptors (63). In the pituitary of human, about 70% of somatotropes have been shown to contain GnRHR immunoreactivities (19). In the rat anterior pituitary, about 38% of GH immunoreactive cells bind biotinylated GnRH (64). These lines of evidence indicate that the regulatory roles of GnRH signals on somatotropes may vary in different species. Considering the early ontogenic appearance of GnRH receptors in somatotropes of ricefield eels and tilapia (62), GnRH signals may play important roles during early development in these species through regulation of somatotropes.

In agreement with the presence of GnRH receptors in somatotropes as stated above, GnRHs have been demonstrated to increase GH release *in vitro* from dispersed rat pituitary cells (4), and to stimulate Gh release in most of teleosts examined, including goldfish (5, 65, 66), grass carp (7), common carp (7, 8), and tilapia (67). In rainbow trout, however, the role of GnRH as a GH-releasing factor is not clear. Some studies reported no effect of GnRH on GH release *in vivo* nor *in vitro* (68, 69), while others observed a stimulatory effect *in vitro* (70). In African catfish, *in vivo* GnRH treatment had no effect on plasma GH levels, which is in agreement with lack of GnRH receptors in somatotropes (63). As in most of the teleosts examined above, both GnRH1 and GnRH3 were shown to stimulate Gh release from dispersed pituitary cells of ricefield eels. In consistency with these *in vitro* studies, GnRH1 and GnRH3 immunoreactive fibers were shown to be in close proximity to Gh cells, even with some co-localizations in the pituitary of ricefield eels. Similarly, GnRH fibers were observed in close association with somatotropes in the pituitary of tilapia (62). Moreover, both GnRH1 and GnRH3 decreased Gh contents in the pituitary of ricefield eels after intraperitoneal injection for 12 h. These lines of evidence suggest that GnRH1 and GnRH3 may directly stimulate Gh release via GnRHR2 but not GnRHR1 in the pituitary of ricefield eels. Admittedly, the involvement of other type(s) of GnRHR in the regulation of somatotropes in ricefield eels could not be excluded at present. As more than two forms of GnRHRs were identified in many teleosts, such as tilapia (2), medaka (22), masu salmon (71), Atlantic cod (27), European eel (28), and European sea bass (24), it is likely that there are additional forms of GnRHRs in the ricefield eel. In the context of different distribution as compared to GnRHR2 in the pituitary of ricefield eels, the physiological relevances of GnRHR1 in the pituitary of ricefield eels are very intriguing and worth further study.

In addition to the stimulation on GH release, GnRHs could also elevate *gh* transcript levels *in vivo* in goldfish pituitary (72), and *in vitro* in goldfish pituitary cells (9) and common carp pituitary fragments (8). In contrast, GnRH1 and GnRH3 had no effects on *gh* mRNA expression in primary cultures of ricefield eel pituitary cells. The differences between Gh release and *gh* mRNA levels in response to GnRH or other signals were also observed in other studies. In cultured pituitary cells of tilapia, GnRH did not affect *gh* mRNA levels while doubled Gh release (6). Direct activation of protein kinase C did not alter *gh* mRNA levels either but increased Gh release in pituitary cells of rat (73) and tilapia (6). Similarly, GnRH did not affect *gh* mRNA levels in cultured pituitary cells of zebrafish (74) and coho salmon (75). It is likely that the effect of GnRH on *gh* transcription may be species dependent. Alternatively, the action of GnRH on *gh* expression is highly time dependent in goldfish pituitary (76) and the lack of response of *gh* transcription to GnRH in those species may need further evaluation (1).

Generally, GnRH peptides bind GnRH receptors and activate multiple signal transduction pathways, mainly protein kinase C (PKC), protein kinase A (PKA), inositol 1,4,5-triphosphate (IP_3_), and calmodulin (77–79). In goldfish, intracellular signal transduction mediating GnRH-stimulation of Gh release involves several signaling pathways including PKC and Ca^2+^ signaling (80–83). In tilapia, the GnRH effect on GH release was also shown to be dependent on PKC (6). Our study showed that the stimulatory effects of GnRH1 and GnRH3 on Gh release were abolished by the PLC, PKC, IP_3_R or VSCC inhibitor, suggesting that both PLC/IP_3_/PKC and Ca^2+^ pathways are involved in the intracellular signal transduction mediating GnRH1 and GnRH3 stimulation on Gh release. GnRHs have been reported to activate adenylate cyclase (AC) to cause cAMP accumulation in human GH-secreting adenomas (84), and to increase cAMP production in GH_3_ cells expressing GnRHRs (85). In contrast, our present study showed that the production of cAMP in the primary culture of pituitary cells of ricefield eels was not stimulated by either GnRH1 or GnRH3. In agreement, the PKA inhibitor did not block the stimulatory effects of GnRH1 or GnRH3 on Gh release from primary culture of pituitary cells. Similarly, neither GnRH2 nor GnRH3 stimulation of Gh release was dependent on cAMP-mediated signaling although activation of AC-cAMP-PKA signaling can increase Gh release from goldfish pituitary cells (86, 87). The PKA pathway seemed not be involved in the GnRH stimulation of GH release in tilapia either (6). These lines of evidence suggest that the cAMP-PKA pathway may not be involved in GnRH-induction of Gh release in teleosts including ricefield eels.

In summary, we identified ricefield eel GnRH1, GnRH2, and GnRH3 from the brain, and GnRHR1 and GnRHR2 from the pituitary. Both GnRH1 and GnRH3 may exert hypophysiotropic roles in ricefield eels. *gnrhr1* was expressed in restricted tissues and predominantly in the pituitary whereas *gnrhr2* expressed broadly in the brain, pituitary, and other peripheral tissues. Moreover, GnRHR1 and GnRHR2 were differentially distributed in the pituitary, with GnRHR2 but not GnRHR1 expressed in somatotropes. GnRH1 and GnRH3 very likely bind to GnRHR2 to stimulate Gh release via PLC/IP_3_/PKC and Ca^2+^ pathways. Results of present study provide novel information on differential roles of multiple GnRH receptors in vertebrates.

## Author contributions

WZ and LZ conceived and designed the research. DC, WY, SH, HY, XC, and JL performed the experiments. DC, SH, LZ, and WZ analyzed data. DC, LZ, and WZ wrote the manuscript.

### Conflict of interest statement

The authors declare that the research was conducted in the absence of any commercial or financial relationships that could be construed as a potential conflict of interest.
