# Supplementary material for: A Type IIb, but Not Type IIa, GnRH Receptor Mediates GnRH-Induced Release of Growth Hormone in the Ricefield Eel
Source: Front Endocrinol (Lausanne). 2018 Nov 30;9:721. doi: 10.3389/fendo.2018.00721 (PMC6283897; doi:10.3389/fendo.2018.00721)
Supplement: Supplementary file 1 [file Table_1.DOC]

**Supplementary Table 1.** Sequences of oligonucleotide primers used in cloning of GnRH and GnRHR.

| **Primer name** | **Sequence (5’-3’)** |
| --- | --- |
| gnrh1-F1  gnrh1-F2  gnrh2-F1  gnrh2-F2  gnrh3-F  gnrh1-R  gnrh2-R  gnrh3-R  gnrhr-F  gnrhr-R1  gnrhr-R2  gnrhr1-5’race-R1  gnrhr1-5’race-R2  gnrhr2-5’race-R1  gnrhr2-5’race-R2  gnrhr1-3’race-F1  gnrhr1-3’race-F2  gnrhr2-3’race-F1  gnrhr2-3’race-F2  dT-AP  AP  GR5P  GR5NP  GR3P  GR3NP | CARCAYTGGTCITAYGG  TAYGGNCTNAGRCCIGGIGG  CARCAYTGGWSICAYGGITGG  CAYGGNTGGTAYCCIGGIGG  CARCAYTGGTCITAYGGITGGYT  CTATATTGCCCAGTGTGTC  CTTCAATATTTCTGCGCCAC  CTGCCACTGCTGCTGAAGATG  GAYGGHATGTGGAAYATYAC  TARATVARDGGRTCRAARCA  CCSARSACGTAGTADGGRATCCARCA  GAATATCTGAGGGACTGACAGCA  CTGATAGCCAGAGGGTTGAGGAT  CTCAGAGCATCCAGCGGGTGTAG  ATGACTCACCTGCTTTATCCCTC  TTGTTCCTGCTGCCACTGGTCAT  TGCATTTGCGGTGTTCAAAGAAT  CCTGCTGGGTTGGTGGTACTGGTTCT  TCTCGCACTCGCTGGCACACATCCTG  GGCCACGCGTCGACTAGTAC(T)_17_  GGCCACGCGTCGACTAGTAC  CGACTGGAGCACGAGGACACTGA  GGACACTGACATGGACTGAAGGAGTA  GCTGTCAACGATACGCTACGTAACG  CGCTACGTAACGGCATGACAGTG |

F: sense primer; R: antisense primer. S=G/C; R=A/G; Y=C/T; W=A/T; V=A/G/C; H=A/C/T; D=A/G/T; N=G+A+T+C.
