# Supplementary material for: A Type IIb, but Not Type IIa, GnRH Receptor Mediates GnRH-Induced Release of Growth Hormone in the Ricefield Eel
Source: Front Endocrinol (Lausanne). 2018 Nov 30;9:721. doi: 10.3389/fendo.2018.00721 (PMC6283897; doi:10.3389/fendo.2018.00721)
Supplement: Supplementary file 2 [file Table_2.DOC]

**Supplementary Table 2.** Sequences of oligonucleotide primers used in RT-PCR, quantitative PCR and construction of expression vectors.

| **Primer name** | **Sequence (5’-3’)** |
| --- | --- |
| gnrhr1-qF  gnrhr1-qR  gnrhr2-qF  gnrhr2-qR  gh-qF  gh-qR  actb-qF  actb-qR  gapdh-qF  gapdh-qR  hprt1-qF  hprt1-qR  gnrhr1-F  gnrhr1-R  gnrhr2-F  gnrhr2-R  gnrhr1-pcDNA3.0-F  gnrhr1-pcDNA3.0-R  gnrhr2-pcDNA3.0-F  gnrhr2-pcDNA3.0-R | TCCTGTTCATCTTTGGACTT  CCTTTCTGAAGCGAATGGTG  GGTGGTCATTGTGCTATCCTTTG  GGAGATAGTGGAGAGGCGTTGC  AAGTCATCCTCCTGCTATCAGTCCT  CTGTCTGCAGAGAGCTCTCAAAGTC  GCAGAGCCTAGACGACCAACTC  GGGTGCGTTTCTTAAACCTAGC  TCACTGCTACCCAGAAGACCG  CTCAGGAATGACCTTGCCCAC  TTGGACAGGACAGAGCGACT  TCATTGGGATGGAGCGGT  GAGAGACCATGGGCCGCTTCAGAAAGGGGATCCAG  GAGAGACTCGAGTTATATGATGCTCTCAGAGCTGGA  GAGAGACCATGGCAGCCCGACATGGTGCACGTC  GAGAGACTCGAGTTAGTTTCTTGCCTGGTTGTTGGT  GAGAGAATTCATGAACACCACTCTGTATGA  GAGACTCGAGTTATATGATGCTCTCAGAGC  GAGAGAATTCATGTCAGGTAACTGGTCTAT  GAGACTCGAGGTTTCTTGCCTGGTTGTTGG |

F: sense primer; R: antisense primer.
