# Supplementary material for: A Type IIb, but Not Type IIa, GnRH Receptor Mediates GnRH-Induced Release of Growth Hormone in the Ricefield Eel
Source: Front Endocrinol (Lausanne). 2018 Nov 30;9:721. doi: 10.3389/fendo.2018.00721 (PMC6283897; doi:10.3389/fendo.2018.00721)
Supplement: Supplementary file 3 [file Data_Sheet_1.PDF]

Supplementary Material

A Type IIb, but Not Type IIa, GnRH Receptor Mediates GnRH-Induced Release of Growth Hormone in the Ricefield Eel

Dong Chen[1], Wei Yang[1], Shiying Han[1], Huiyi Yang[1], Xin Cen[1], Jiang Liu[1], Lihong Zhang[1*], and Weimin Zhang[1,2*]

***Correspondence:**
Lihong Zhang, zhlih@mail.sysu.edu.cn;
Weimin Zhang, lsszwm@mail.sysu.edu.cn

SUPPLEMENTARY DATA

Sequence and Phylogeny Analysis

The potential open reading frames (ORF) of ricefield eel prepro-GnRHs and GnRHRs were analyzed and translated into the corresponding amino acids using DNAtools (Ft. Colins, USA). The putative seven-transmembrane domains of ricefield eel GnRHRs were predicted using TMHMM Server v. 2.0. For phylogeny analysis, the publicly available full-length amino acid sequences of prepro-GnRHs and GnRHRs were obtained from NCBI databases via the accession numbers provided in the supplementary material (Supplementary Figures 2 and 6), except the full-length sequence of sea bass GnRHR2c, which was obtained from Moncaut et al. (2005). Including sequences identified in the present study, total of 35 sequences for prepro-GnRHs and 47 sequences for GnRHRs were aligned using Clustalx 1.83 program, respectively. Phylogenetic trees were constructed by the neighbor-joining method using MEGA 7.0 software, based on the aligned amino acid sequences of whole prepro-GnRHs or GnRHRs. Bootstrap values (%) were calculated from 1,000 replications to estimate the robustness of internal branches. The fruit fly (*Drosophila melanogaster*) GnRHR homolog was used as an out-group to root the tree of GnRHRs.

Measurement of Intracellular cAMP in Primary Pituitary Cells of Ricefield Eels

To assay the effects of GnRH1 and GnRH3 on cAMP production in primary pituitary cells of intersexual ricefield eels, the dispersed pituitary cells were placed evenly into 24-well plates (Nunc, Denmark) at approximately 1×10^6 cells/mL per well with DMEM (Gibco, MA, USA) containing 10% FBS (Gibco) and cultured at 28℃ with 5% CO_2. After pre-incubation for 24 h, the medium was changed, and the cells were starved in DMEM without FBS for 12 h before treatments. Prior to each experiment, cells were washed with DMEM and allowed to rest for 1 h. The pituitary cells were subsequently treated with GnRH1 (100 nM), GnRH3 (100 nM), or forskolin (10 μM) for 4 h. The working solutions of GnRH1, GnRH3, and forskolin were diluted from stocks to the desired concentrations with DMEM, containing 0.1% DMSO. DMEM with 0.1% DMSO was used as the vehicle control. Each treatment was repeated in four wells. After

treatment, the pituitary cells per well were collected and homogenized in 200 µL 0.1 M HCl. After centrifugation at 1000 × g for 5 min at room temperature, the supernatants were collected and the amount of cAMP in pituitary cells was quantified with a Monoclonal Anti-cAMP Antibody Based Direct cAMP ELISA Kit (catalog number 80203, NewEast Biosciences, Inc., PA, USA) by following manufacturer's instruction. Results are expressed as measured cAMP concentrations in pituitary cell homogenates. The experiments were repeated twice, and similar results were obtained.

References

Moncaut N, Somoza G, Power DM, Canário AV. Five gonadotrophin-releasing hormone receptors in a teleost fish: isolation, tissue distribution and phylogenetic relationships. *J Mol Endocrinol* (2005) 34(3):767–79. doi:10.1677/jme.1.01757
