# Supplementary material for: A Type IIb, but Not Type IIa, GnRH Receptor Mediates GnRH-Induced Release of Growth Hormone in the Ricefield Eel
Source: Front Endocrinol (Lausanne). 2018 Nov 30;9:721. doi: 10.3389/fendo.2018.00721 (PMC6283897; doi:10.3389/fendo.2018.00721)
Supplement: Supplementary file 4 [file Data_Sheet_2.PDF]

GnRH1:

```
          ATTCACTAAGGACTGCAGATGAGAACAAATCAGGGAAGCTGCCAGCCTT ATG CAC AGA AGA ATG   64
                                                            M   H   R   R   M      5
ACT GTG AAA ACC ATG GCA CTG TGG CTG CTG CTT GTG GGG ACA CTG GTG CCA CAG CTC TAC  124
T   V   K   T   M   A   L   W   L   L   L   V   G   T   L   V   P   Q   L   Y     25
TGT CAG CAC TGG TCG TTT GGA CTG AGT CCA GGA GGG AAG AGG GAA CTG AAC AGC CTG TCA  184
C   Q   H   W   S   F   G   L   S   P   G   G   K   R   E   L   N   S   L   S     45
GAC ACA CTG GGC AAT ATA GTT GAA GGA TTT CCA CAT GTG GAC GCA CCC TGC AGC ATT TTG  244
D   T   L   G   N   I   V   E   G   F   P   H   V   D   A   P   C   S   I   L     65
CGT TGT GCA GAG GAA TTG CCT TTT GCG AGT ATT TAC AGA ATG AAA GGA TTT CTT GAC GGT  304
R   C   A   E   E   L   P   F   A   S   I   Y   R   M   K   G   F   L   D   G     85
GTC ACT GAC ACG GAA AAC GGA CAC AGA ACA TAC AAA AAA TGA TTATTGTTTGAGTCTACAATAAA  369
V   T   D   T   E   N   G   H   R   T   Y   K   K   *                             98
TTATGATATGAGC(A)n                                                                 382
```

GnRH2:

```
                                                                       GAGAATCTT    9
TACAGTAAAACACCTGTGAAACCGTGAGATACCTTTAGAACTGGTCTACTGAGGGAAAAGCAGAGTGGACTTCTGGAGA   88
GACGTTAAGGGACACCAGTGCCAAAAAAGTTGAGTTTAGACTAAGATTTCCTAAAGCAGGTCAAACAGCAGGTGGAAAT  167
ATG ATT CGG CTG GTC TTG CTG CTG GGG CTC CTT CTA TGT GTG GGG GTT CAG CTG TCC GGC  227
M   I   R   L   V   L   L   L   G   L   L   L   C   V   G   V   Q   L   S   G     20
GCC CAG CAC TGG TCC CAT GGC TGG TAT CCT GGT GGC AAG AGG GAG CTG GAC TCT TTC CCC  287
A   Q   H   W   S   H   G   W   Y   P   G   G   K   R   E   L   D   S   F   P     40
ACA TCA GAG ATT TCA GGG GAG ATT AAT CTG TGT GAG GCA GGG GAA TGC AGC TAC CTG AGA  347
T   S   E   I   S   G   E   I   N   L   C   E   A   G   E   C   S   Y   L   R     60
CCC CAG AGG AGG AGC ATA CTC AGA AGC ATT CTT TTG AAT GTA TTA GCC AGA GAG CTC CAG  407
P   Q   R   R   S   I   L   R   S   I   L   L   N   V   L   A   R   E   L   Q     80
AAG AGA AAG TGA CAGCTTTCCACCCTACACTGCTTTTCTTCCTAGTGGCCCTCTTTATCATGGCATGAATCAACT  482
K   R   K   *                                                                     83
TCATGCCGATCCTCTGATCTTGTGCATTTGTTCAGTAAAACTGTTTTCCATGGTTTCTCTTTGTGGCGCAGAAATATTG  561
AAGTTACAATATCCCATAATAAAGTATCTATTTTGATATT(A)n                                     601
```

GnRH3:

```
 TTCTCATTGAATTTGCTCAATGTTAATTAAGTTAGAATTACTCTTATTAATTACCATCTGAAGTTTAGTTTACTAATG   78
TTGAGTAACCCTTGATCTGTGGAGCTGTGAATGAGGATACAGTGACTTTTTCTTTTACAACTCTGGCTTCAGTGCAGCT  157
CTA ATG GAG GCA AGC AGC AGA GTG ACG GTG CAG GTG TTG TTG TTG GCG TTG GTG GTT CAG  217
    M   E   A   S   S   R   V   T   V   Q   V   L   L   L   A   L   V   V   Q     19
GTC ACC CTG TCC CAG CAC TGG TCC TAC GGG TGG CTA CCA GGT GGA AAA AGA AGT GTG GGA  277
V   T   L   S   Q   H   W   S   Y   G   W   L   P   G   G   K   R   S   V   G     39
GAG CTT GAG GCA ACC ATC AGG ATG ATA GGC ACA GGA GGA GTG GTG ACT CTT CCC GAT GAG  337
E   L   E   A   T   I   R   M   I   G   T   G   G   V   V   T   L   P   D   E     59
GCG AGC GCC CCA ATC CAA CAG AGA CTT AGA CCA TAC AAT GTT ATT AAT GAT GAT TCC AGT  397
A   S   A   P   I   Q   Q   R   L   R   P   Y   N   V   I   N   D   D   S   S     79
CAT TTT GAC CAA AGG AAA AGG TTC TCT AAT AAA TGA AGAGCTGCAAAACCAAAAGGAAAAGAAAAGA  464
H   F   D   Q   R   K   R   F   S   N   K   *                                     90
AACGCCACTGTACTTTCATCTTCAGCAGCAGTGGCAGACTCCCTAAATTCAGGATGATGACTCCAACAAATCACCACAT  543
CTGTTACACAGTTATCAATTAAATGTCTGTAACATTTACATCCATGCAAAATTGTAATAAAGTTTTTGGAATCTG(A)n  618
```

Supplementary Figure 1. Nucleotide and deduced amino acid sequences of ricefield eel GnRH1, GnRH2, and GnRH3 precursors. The nucleotides (upper row) and amino acids (lower row) are numbered on the right-hand side of the sequences. The signal

peptide, GnRH decapeptide and GnRH-associated peptide regions are single underlined, boxed, and double underlined, respectively. The asterisk (*) indicates the stop codon. The nucleotides corresponding to the polyadenylation signal (AATAAA) are shown in bold.
