# Supplementary material for: A Type IIb, but Not Type IIa, GnRH Receptor Mediates GnRH-Induced Release of Growth Hormone in the Ricefield Eel
Source: Front Endocrinol (Lausanne). 2018 Nov 30;9:721. doi: 10.3389/fendo.2018.00721 (PMC6283897; doi:10.3389/fendo.2018.00721)
Supplement: Supplementary file 5 [file Data_Sheet_3.PDF]

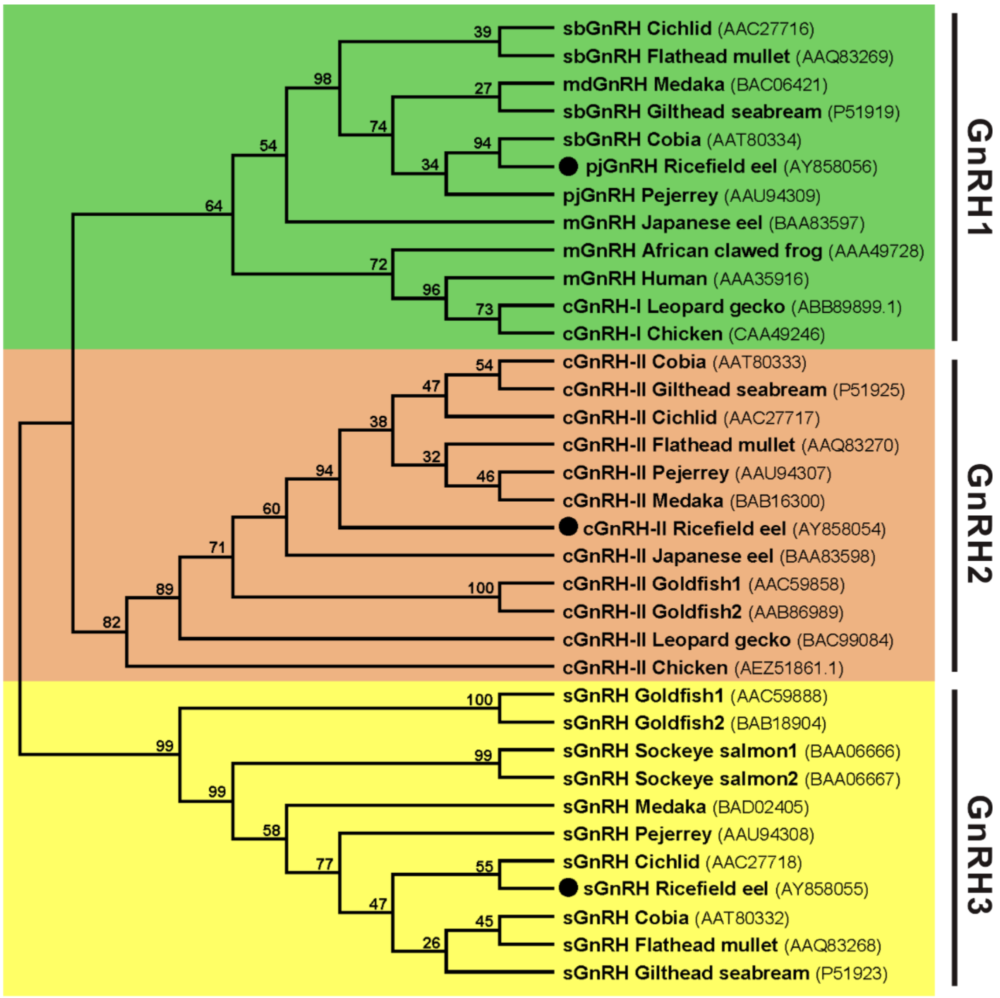

Supplementary Figure 2. Phylogenetic analysis of ricefield eel prepro-GnRHs with those of other vertebrates. Vertebrate GnRH1, GnRH2, and GnRH3 were shown in the green, orange, and yellow background, respectively. The unrooted tree was constructed by the neighbor-joining method using MEGA 7.0 software, based on the alignments of the amino acid sequences of whole prepro-GnRHs using Clustalx 1.83 program. The number shown at each branch node indicates the bootstrap value (%) estimated by 1,000 times replications. The protein sequences were downloaded from *Entrez* (NCBI). Human, *Homo sapiens*; Leopard gecko, *Eublepharis macularius*; African clawed frog, *Xenopus laevis*; Chicken, *Gallus gallus*; Medaka, *Oryzias latipes*; Pejerrey, *Odontesthes bonariensis*; Goldfish, *Carassius auratus*; Gilthead seabream, *Sparus aurata*; Sockeye salmon, *Oncorhynchus nerka*; Cichlid, *Haplochromis burtoni*; Flathead mullet, *Mugil cephalus*; Japanese eel, *Anguilla japonica*; Ricefield eel, *Monopterus albus*; Cobia, *Rachycentron canadum*.
