# Supplementary material for: A Type IIb, but Not Type IIa, GnRH Receptor Mediates GnRH-Induced Release of Growth Hormone in the Ricefield Eel
Source: Front Endocrinol (Lausanne). 2018 Nov 30;9:721. doi: 10.3389/fendo.2018.00721 (PMC6283897; doi:10.3389/fendo.2018.00721)
Supplement: Supplementary file 6 [file Data_Sheet_4.PDF]

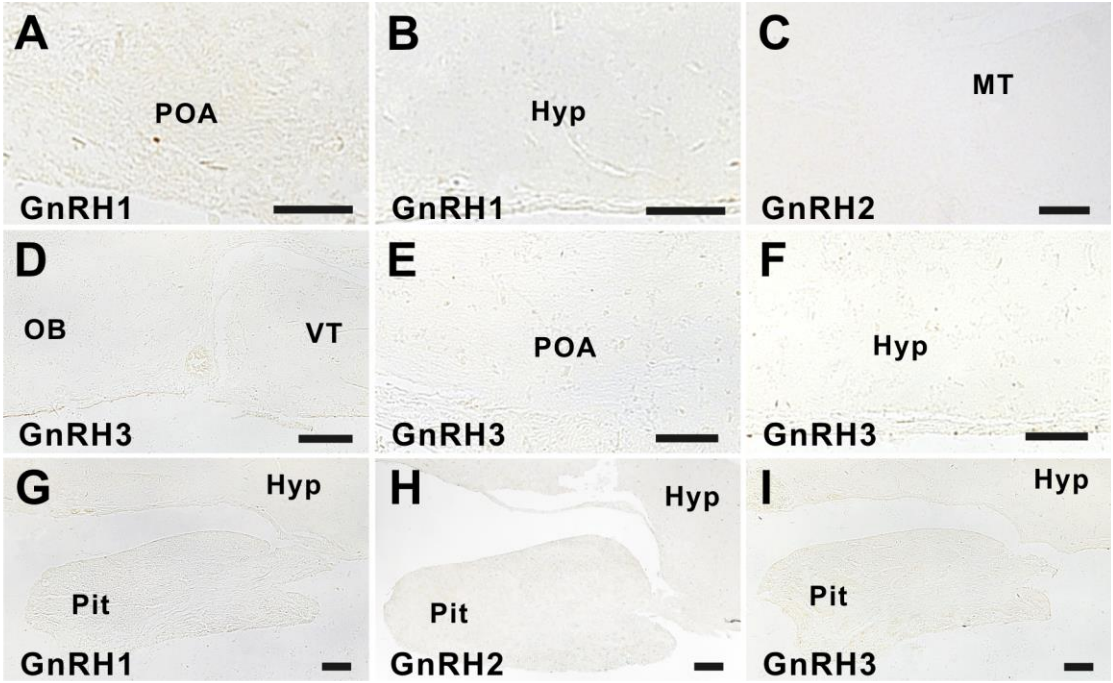

Supplementary Figure 3. Blockage of immunoreactive GnRH1, GnRH2, and GnRH3 in the brain and pituitary by the pre-adsorption of antiserum with an excess of the corresponding synthetic ricefield eel GnRH peptide. Sagittal sections of the brain together with the pituitary gland of ricefield eels were immunoreacted with primary antibodies, the pre-adsorbed rabbit polyclonal antibody AS-691 [1:7000 dilution; **(A,B,G)**] by 0.25 μg/mL ricefield eel GnRH1, the pre-adsorbed rabbit polyclonal antibody 675 [1:2000 dilution; **(C,H)**] by 0.25 μg/mL ricefield eel GnRH2, or the pre-adsorbed mouse monoclonal antibody LRH13 [1:2000 dilution; **(D,E,F,I)**] by 0.25 μg/mL ricefield eel GnRH3. After incubation with primary antibodies for 40 h at 4°C, the sections were then exposed to the secondary antibody, HRP-conjugated goat anti-rabbit or anti-mouse IgG (H+L) (1:1000 dilution; Beyotime, Shanghai, China), and finally visualized with 3,3'-diaminobenzidine (DAB) solution, mounted, and digitally photographed with a Nikon Eclipse Ni-U microscope (Japan). OB, olfactory bulbs; VT, ventral telencephalon; POA, preoptic area; MT, midbrain tegmentum; Hyp, hypothalamus; Pit, pituitary. Scale bar = 50 μm.
