# Supplementary material for: A Type IIb, but Not Type IIa, GnRH Receptor Mediates GnRH-Induced Release of Growth Hormone in the Ricefield Eel
Source: Front Endocrinol (Lausanne). 2018 Nov 30;9:721. doi: 10.3389/fendo.2018.00721 (PMC6283897; doi:10.3389/fendo.2018.00721)
Supplement: Supplementary file 7 [file Data_Sheet_5.PDF]

```
                                             GAAAAGGGCGCAGTGCGCAGCAGAGGAGTCATAGCAGTCC      40
ACCTAAAACAAACGAATGGCGACACCTACTTGGCTCCTGCTGTGATTTTAAATACTTAGACCTCGACACCTGCGCAACC     119
CACATCCTTCTCCACTGCATATACTGCTGCTCTTCGTGGTGGATGAGCGCAATGATTCCTCCTCTTCAAACACCAGAAA     198
ATG AAC ACC ACT CTG TAT GAC TCT GCA GTG ACT ATG TAT CAG CTG ATA GCA GGT CAA CTG     258
 M   N   T   T   L   Y   D   S   A   V   T   M   Y   Q   L   I   A   G   Q   L       20
                 N-terminal extracellular domain
AAT GCC AGC TGC AAC TTC TCC TTG TCC CCT TCC AAC TGG TCA GCA GAG GCC GAA GGT CTG     318
 N   A   S   C   N   F   S   L   S   P   S   N   W   S   A   E   A   E   G   L       40

CAG CTG CCC ACA TTC ACC ACA GCG GCC AAA GTC AGA GTG ATC ATT ACC TTC ATT CTC TGT     378
 Q   L   P   T   F   T   T   A   A   K   V   R   V   I   I   T   F   I   L   C       60
                                                          TMD 1
GGC ATC TCT GCC TTT TGC AAC CTG GCT GTG CTA TGG GCG GCA CAC AGC AGG AAG CGT AAA     438
 G   I   S   A   F   C   N   L   A   V   L   W   A   A   H   S   R   K   R   K       80
                                                              ICL 1
TCC CAT GTC AGG GTG TTG ATA ATC AAT TTG ACG GTG GCT GAT CTG CTG GTG ACC TTC ATT     498
 S   H   V   R   V   L   I   I   N   L   T   V   A   D   L   L   V   T   F   I      100
                                                 TMD 2
GTG ATG CCT GTG GAT GCT GCG TGG AAC ATC ACA GTT CAG TGG CTC GCT GGC GAC TTT GCC     558
 V   M   P   V   D   A   A   W   N   I   T   V   Q   W   L   A   G   D   F   A      120
                                                     ECL 1
TGC AGG CTA CTG ATG TTT CTT AAG CTG CAG GCA ATG TAC TCC TGC GCA TTT GTC ACT GTG     618
 C   R   L   L   M   F   L   K   L   Q   A   M   Y   S   C   A   F   V   T   V      140
                                         TMD 3
GTG ATC AGT CTG GAT AGG CAG TCA GCC ATC CTC AAC CCT CTG GCT ATC AGT AAA GCC AGA     678
 V   I   S   L   D   R   Q   S   A   I   L   N   P   L   A   I   S   K   A   R      160
                                                 ICL 2
AAG AGG AAC AGA TTC ATG CTG ACT GTG GCG TGG GGC ATG AGT ACA GTG CTG TCA GTC CCT     738
 K   R   N   R   F   M   L   T   V   A   W   G   M   S   T   V   L   S   V   P      180
                                                 TMD 4
CAG ATA TTC CTT TTT CAC AGC TTG ACC ATT ATC CAT CCT GAA GAC TTC ACT CAG TGT ACA     798
 Q   I   F   L   F   H   S   L   T   I   I   H   P   E   D   F   T   Q   C   T      200
                                                     ECL 2
ACT CGT GGA AGT TTT GTC ACT CCC TGG CAT GAG ACG GCC TAC AAC ATG TTC ACT TTT TCT     858
 T   R   G   S   F   V   T   P   W   H   E   T   A   Y   N   M   F   T   F   S      220

TGC TTG TTC CTG CTG CCA CTG GTC ATC ATG ATC ACT TGT TAC ACC AGG ATC TTC TGT GAA     918
 C   L   F   L   L   P   L   V   I   M   I   T   C   Y   T   R   I   F   C   E      240
                                     TMD 5
GTC TCC AAA CGC CTG AAA AAG GAT AAC TGG CCC TCA AAT GAA GTG CAT TTG CGG TGT TCA     978
 V   S   K   R   L   K   K   D   N   W   P   S   N   E   V   H   L   R   C   S      260
                                         ICL 3
AAG AAT AAC ATC CCC AGA GTC CGG ATG AAA GCT CTG AAG ATG AGT ATT GTA ATC GTT TTG    1038
 K   N   N   I   P   R   V   R   M   K   A   L   K   M   S   I   V   I   V   L      280

TCT TTC GTT ATC TGC TGG ACT CCA TAC TAC CTG CTG GGT TGG TGG TAC TGG TTC TTT CCT    1098
 S   F   V   I   C   W   T   P   Y   Y   L   L   G   W   W   Y   W   F   F   P      300
                                     TMD 6
GAT GAT CTG GAA GAG AAG GTC TCG CAC TCG CTG GCA CAC ATC CTG TTC ATC TTT GGA CTT    1158
 D   D   L   E   E   K   V   S   H   S   L   A   H   I   L   F   I   F   G   L      320
                 ECL 3
GTC AAC GCC TGC CTG GAC CCA GTC ATC TAT GGC ATG TTC ACC ATT CGC TTC AGA AAG GGG    1218
 V   N   A   C   L   D   P   V   I   Y   G   M   F   T   I   R   F   R   K   G      340
                          TMD 7
ATC CAG AGG TAT TAC TGC AAT GCC GCC ACA TCA TCG AAC CTG GAT AAT AAT ACT GTT ATA    1278
 I   Q   R   Y   Y   C   N   A   A   T   S   S   N   L   D   N   N   T   V   I      360
                    C-terminal intracellular domain
ACT AGC TCT TTA ATT TGT GCT GCC AAT TCT TTG CTG CTG AAA AGA GAG GTG AGT GCC ACC    1338
 T   S   S   L   I   C   A   A   N   S   L   L   L   K   R   E   V   S   A   T      380

AGC CAG GAG GCA TTC ATG CTG TGC AGA GAT AAT CAC AGC AAA GCA GGA AGC AGC TTT TTA    1398
 S   Q   E   A   F   M   L   C   R   D   N   H   S   K   A   G   S   S   F   L      400

ACA GAA AGA GAT GTA AAC CAG TCC AGC TCT GAG AGC ATC ATA TAAAGAGATCAGTTTTACTTCTG    1463
 T   E   R   D   V   N   Q   S   S   S   E   S   I   I   *                          414
CTGTACATCTATACTTCAGATTTATTCAGTGTCCATAGATCTCGTGTGCAGGGCTTTATTTCTTAATACTGCTCTTCGT    1542
AGCTGTTTATAAATAACAAAAATAGTATTCAATGTGATTTGGAGTGTCTGCTAGAGGTAACATTACAATTGCAAATGGA    1621
AGCGCACAGCAACACATTCAATTTGTGTTCAGATTTCTTATGCAAAGCCATTTCTGTCCCTGTACTGATGTGTAGCAGT    1700
```

```
AGGGTCCCTGTGGGGTTTTGTTTGAACCTTAAGCCCCTTCCTTCCTGGTGACTGACTGTTAATACCAGGGAAAAATAAA     1779
AGTATATAAAAAAAGCCAAAAAAAAAAAAAAAAAAAAAAAAAA                                        1822
```

Supplementary Figure 4. Nucleotide and deduced amino acid sequences of ricefield eel GnRHR1. The seven putative transmembrane domains (TMD 1-7) were predicted using the TMHMM Server v. 2.0 (http://www.cbs.dtu.dk/services/TMHMM/) and indicated in gray. The nucleotides (upper row) and amino acids (lower row) were numbered on the right-hand sides of the sequences. The translation stop codon (TAA) was indicated by an asterisk (*) and a potential polyadenylation signal (AATAAA) in the 3'-untranslated region was underlined in bold. ICL, intracellular loop; ECL, extracellular loop.
