# Supplementary material for: A Type IIb, but Not Type IIa, GnRH Receptor Mediates GnRH-Induced Release of Growth Hormone in the Ricefield Eel
Source: Front Endocrinol (Lausanne). 2018 Nov 30;9:721. doi: 10.3389/fendo.2018.00721 (PMC6283897; doi:10.3389/fendo.2018.00721)
Supplement: Supplementary file 8 [file Data_Sheet_6.PDF]

```
                                                           GAAAGCAGAGACAC     14
AATCCTGAACTGGTTTGGACTACACCAGGTTCTGTGTCTCAGACTGGGATCTGCAGGTTCTTCAGGGTTCTCTAAATTT     93
TGGACTGAAGCTTGAACATGAGGAGAGGAGAACGCAGTCCAACCTGAGGAGAAGAAAAGATTCAGAGCTGCTGAGCAGG    172
ATG TCA GGT AAC TGG TCT ATC CTG AGA CTG TCA CCA CTG GCT CCA CCC ACC AGC TCT GTA    232
 M   S   G   N   W   S   I   L   R   L   S   P   L   A   P   P   T   S   S   V      20
                       N-terminal extracellular domain
GCC TTA CTT CCC AAC ACC TCC CAG TTC CCC CCA CTC TCA GAC TGG GAG GCC CCC AGT TTC    292
 A   L   L   P   N   T   S   Q   F   P   P   L   S   D   W   E   A   P   S   F      40

ACT CGG GCT GCT CAG TTC CGT GTT GCA GCC ACC TTG GTC CTC TTC CTT TTC GCT GCC TGC    352
 T   R   A   A   Q   F   R   V   A   A   T   L   V   L   F   L   F   A   A   C      60
                                                       TMD 1
AGT AAC CTG GCT TTG TTG GTC AGT GTG TGG TGT GGG CGT GGA CGG CAG CTG GCA TCT CAT    412
 S   N   L   A   L   L   V   S   V   W   C   G   R   G   R   Q   L   A   S   H      80
                                                            ICL 1
CTG CGA CCG CTG ATG CTG AGC CTG GTG GCA GCT GAC CTG ATG ATG ACA TTT GTG GTG ATG    472
 L   R   P   L   M   L   S   L   V   A   A   D   L   M   M   T   F   V   V   M     100
                                              TMD 2
CCT CTG GAT GCG GTG TGG AAC ATC ACA GTT CAG TGG TAT GGA GGA GAC GTG CTC TGT AAG    532
 P   L   D   A   V   W   N   I   T   V   Q   W   Y   G   G   D   V   L   C   K     120
                                                   ECL 1
CTG CTC TGC TTC CTA AAG CTG TTC GCC ATG CAC GCA TCT GCC TTC ATC CTT GTT GTC ATC    592
 L   L   C   F   L   K   L   F   A   M   H   A   S   A   F   I   L   V   V   I     140
                                        TMD 3
AGC CTT GAC CGT CAG CAC GCC ATA CTA CAC CCG CTG GAT GCT CTG AGT GCA CAC TGC AGG    652
 S   L   D   R   Q   H   A   I   L   H   P   L   D   A   L   S   A   H   C   R     160
                                              ICL 2
AAT CGA CGC ATG CTG CTA GTG GCC TGG AGC CTC AGT CTG CTG CTT GCA TCA CCA CAG CTG    712
 N   R   R   M   L   L   V   A   W   S   L   S   L   L   L   A   S   P   Q   L     180
                                               TMD 4
TTC ATC TTC AGA GCG GTC CGG GTT GAG GCT GTC GAC TTC ACT CAG TGT GCA ACT CAT GGC    772
 F   I   F   R   A   V   R   V   E   A   V   D   F   T   Q   C   A   T   H   G     200
                                             ECL 2
AGC TTT GGC CAC CGC TGG CAG GAG ACG GTT TAC AAC ATG TTC CAC TTC ACC ACG CTG TAC    832
 S   F   G   H   R   W   Q   E   T   V   Y   N   M   F   H   F   T   T   L   Y     220

ATC ATC CCC CTG CTG GTG ATG AGC TGC TGC TAC AGC CGC ATC CTT CTG CAC ATC CAC CAG    892
 I   I   P   L   L   V   M   S   C   C   Y   S   R   I   L   L   H   I   H   Q     240
                                 TMD 5
CAG CAT CTG AGG GAT AAA GCA GGT GAG TCA TAC CTG CGT CGC AGT GGC ACT GAC ATC ATC    952
 Q   H   L   R   D   K   A   G   E   S   Y   L   R   R   S   G   T   D   I   I     260
                                         ICL 3
CCC AAA GCT CGG ATG AAG ACT CTG AAG ATG ACG GTG GTC ATT GTG CTA TCC TTT GTA GTG   1012
 P   K   A   R   M   K   T   L   K   M   T   V   V   I   V   L   S   F   V   V     280

TGC TGG ACT CCA TAC TAC CTG CTG GGG ATC TGG TAC TGG TTC CAG CCC GAC ATG GTG CAC   1072
 C   W   T   P   Y   Y   L   L   G   I   W   Y   W   F   Q   P   D   M   V   H     300
                              TMD 6
GTC ACA CCT GAG TAT ATC CAC CAC GCC CTC TTT GTG TTC GGG AAC CTG AAC ACC TGC TGT   1132
 V   T   P   E   Y   I   H   H   A   L   F   V   F   G   N   L   N   T   C   C     320
          ECL 3
GAC CCA GTC ATC TAT GGC TTC TAC ATG CCA TCC TTC AGG GCT GAC CTC GCC GCC TGC TGT   1192
 D   P   V   I   Y   G   F   Y   M   P   S   F   R   A   D   L   A   A   C   C     340
          TMD 7
CAC CGG ACA ACA AGC AAC GCC TCT CCA CTA TCT CCG GAG CAG TTC TCC GGC AGG CAG GGT   1252
 H   R   T   T   S   N   A   S   P   L   S   P   E   Q   F   S   G   R   Q   G     360
                        C-terminal intracellular domain
CAT CAC AGT GGG AAG TTG CAC CCT GCC ACC AAC AAC CAG GCA AGA AAC TGACGCTGACCAGTC   1315
 H   H   S   G   K   L   H   P   A   T   N   N   Q   A   R   N   *                 376
AACGGCCAGGAGTTAATCAATGTCATTGTCAGCTTGTGAAGACCTGAACTGGGCCTGCAGGAGGCGGACCAGGACTCTG   1394
AGTCTGCCCCCTGGTGGACAAACACCACAAACCGCAGCTTTAACAGGACATTAACATTTTTCTATGATTGTCTCTGTA   1473
AAAGGATAAAATCGCTGATCATTACTTTATATAAAATTGTTTTTAAAAAAAAAAAAAAAAA                    1534
```

Supplementary Figure 5. Nucleotide and deduced amino acid sequences of ricefield eel GnRHR2. The seven putative transmembrane domains (TMD 1-7) were predicted using the TMHMM Server v. 2.0 (http://www.cbs.dtu.dk/services/TMHMM/) and

indicated in gray. The nucleotides (upper row) and amino acids (lower row) were numbered on the right-hand sides of the sequences. The translation stop codon (TGA) was indicated by an asterisk (*) and the potential polyadenylation signal (ATAAA) in the 3'-untranslated region was underlined in bold. ICL, intracellular loop; ECL, extracellular loop.
