# Supplementary material for: A Type IIb, but Not Type IIa, GnRH Receptor Mediates GnRH-Induced Release of Growth Hormone in the Ricefield Eel
Source: Front Endocrinol (Lausanne). 2018 Nov 30;9:721. doi: 10.3389/fendo.2018.00721 (PMC6283897; doi:10.3389/fendo.2018.00721)
Supplement: Supplementary file 9 [file Data_Sheet_7.PDF]

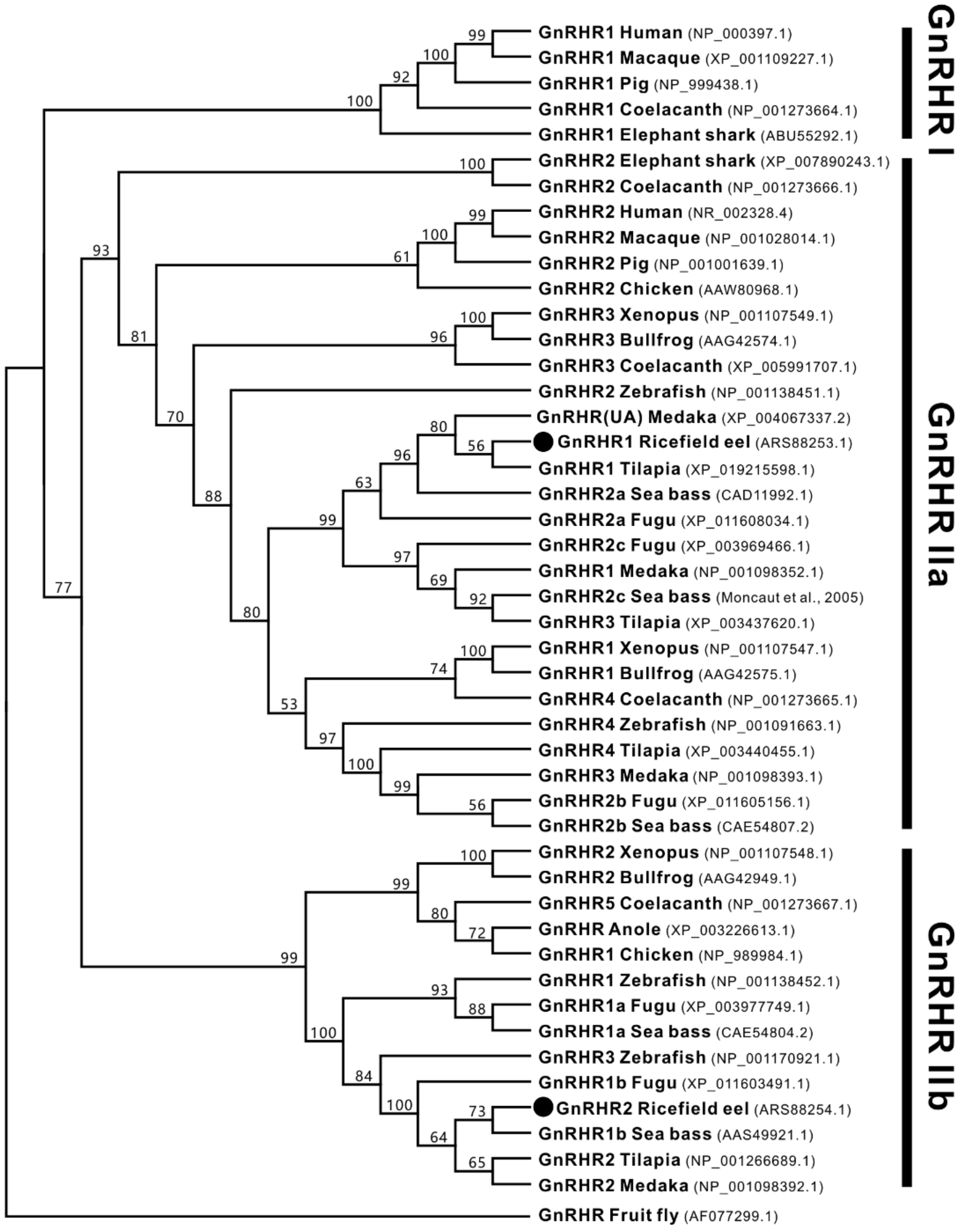

Supplementary Figure 6. Phylogenetic analysis of ricefield eel GnRHRs with those of other vertebrates. All vertebrate GnRHRs were classified as GnRHR I, GnRHR IIa and GnRHR IIb. The phylogenetic tree was constructed by the neighbor-joining method using MEGA 7.0 software, based on the alignments of the amino acid sequences of whole GnRHRs using Clustalx 1.83 program. The number shown at each branch node indicates the bootstrap value (%) estimated by 1,000 times replications. The GnRHR homolog identified in the fruit fly *Drosophila melanogaster* was used as an out-group to root the tree. The protein sequences were downloaded from *Entrez* (NCBI) except the full-length sequence of sea bass GnRHR2c was

obtained from Moncaut et al. (J Mol Endocrinol, 2005, 34:767–79). Human, *Homo sapiens*; Macaque, *Macaca mulatta*; Pig, *Sus scrofa*; Anole, *Anolis carolinensis*; Bullfrog, *Rana catesbeiana*; Xenopus, *Xenopus tropicalis*; Chicken, *Gallus gallus*; Medaka, *Oryzias latipes*; Tilapia, *Oreochromis niloticus*; Sea bass, *Dicentrarchus labrax*; Fugu, *Takifugu rupribes*; Zebrafish, *Danio rerio*; Ricefield eel, *Monopterus albus*; Coelacanth, *Latimeria chalumnae*; Elephant Shark, *Callorhinchus milii*; Fruit fly, *Drosophila melanogaster*; UA, unannotated.
