# Supplementary material for: A Type IIb, but Not Type IIa, GnRH Receptor Mediates GnRH-Induced Release of Growth Hormone in the Ricefield Eel
Source: Front Endocrinol (Lausanne). 2018 Nov 30;9:721. doi: 10.3389/fendo.2018.00721 (PMC6283897; doi:10.3389/fendo.2018.00721)
Supplement: Supplementary file 10 [file Data_Sheet_8.PDF]

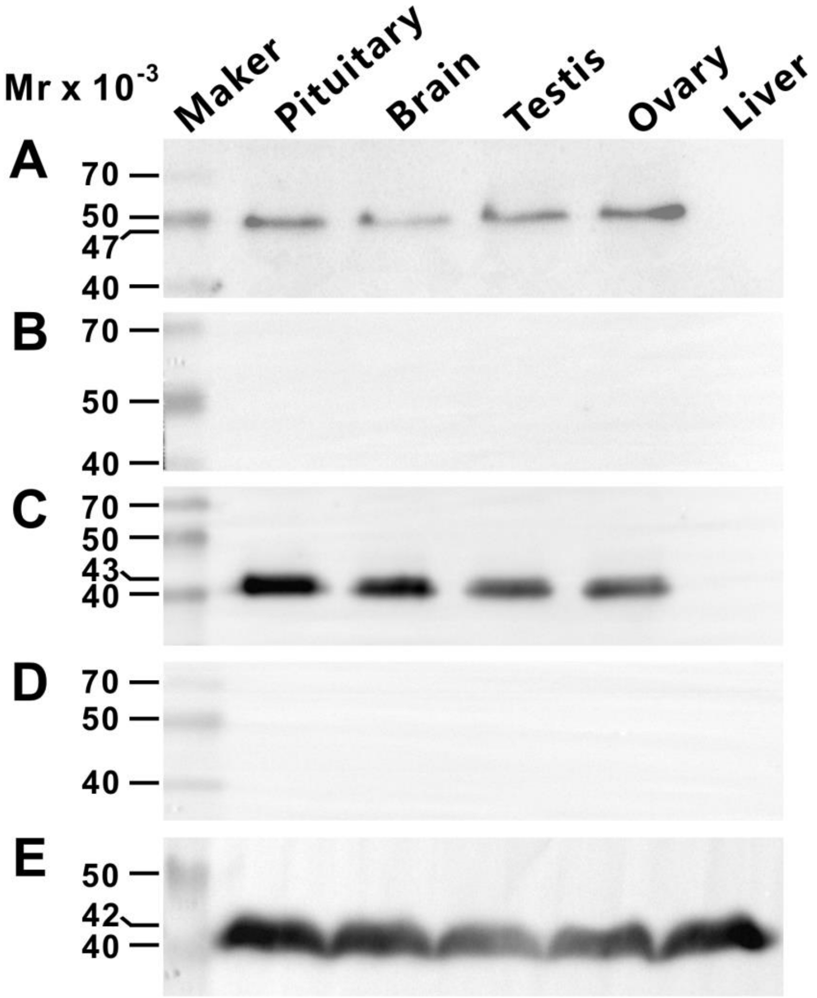

Supplementary Figure 7. Specificities of anti-GnRHR1 **(A)** and anti-GnRHR2 **(C)** antisera against tissue extracts of ricefield eels as determined by Western blot analysis. The tissue extracts (100 µg) from the pituitary of mixed sexes, the brain, liver, and ovary of female fish, and the testis of male fish were separated on 12% SDS-PAGE gels, transferred to polyvinylidenefluoride membranes, and then immunoreacted with the rabbit anti-GnRHR1 antiserum (1:2000 dilution； **A**), the anti-GnRHR1 antiserum pre-absorbed by 10 ug/mL recombinant GnRHR1 expressed in transfected COS-7 cells **(B)**, the mouse anti-GnRHR2 antiserum (1:1000 dilution; **C**), the anti-GnRHR2 antiserum pre-absorbed by 10 ug/mL recombinant GnRHR2 expressed in transfected COS-7 cells **(D)**, or the mouse Actb monoclonal antibody (1:2000 dilution; 60008-1-Ig, ProteinTech Group, Inc., IL, USA; **E**). The secondary antibody was 1:1000 diluted horseradish peroxidase (HRP)-conjugated goat anti-rabbit or anti mouse IgG (H+L) (Beyotime). The blots were visualized using the BeyoECL Plus kit (Beyotime).
