# Supplementary material for: A Type IIb, but Not Type IIa, GnRH Receptor Mediates GnRH-Induced Release of Growth Hormone in the Ricefield Eel
Source: Front Endocrinol (Lausanne). 2018 Nov 30;9:721. doi: 10.3389/fendo.2018.00721 (PMC6283897; doi:10.3389/fendo.2018.00721)
Supplement: Supplementary file 11 [file Data_Sheet_9.PDF]

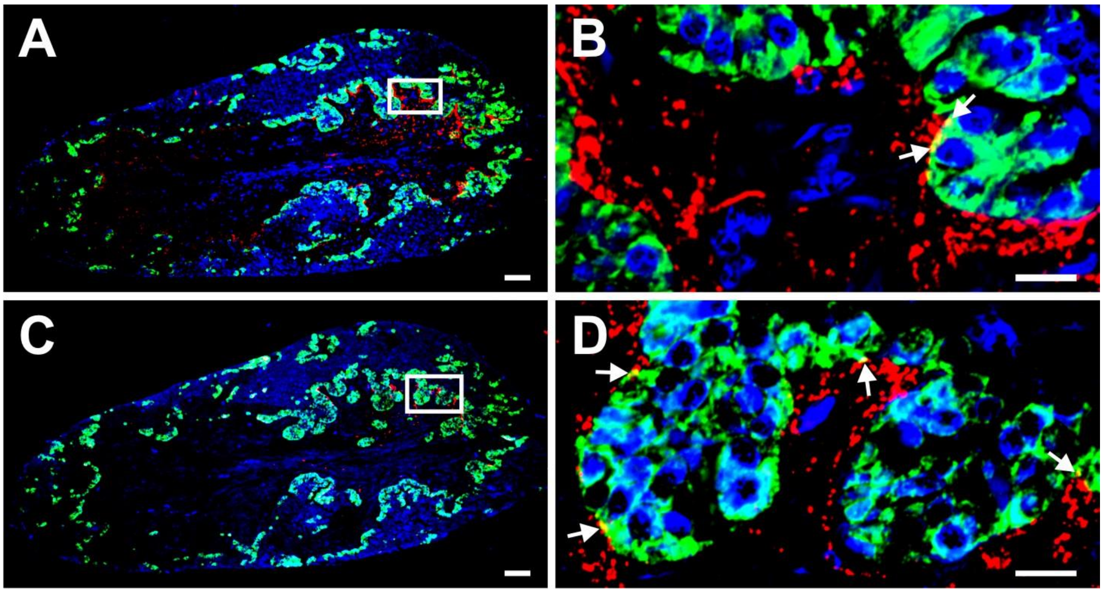

Supplementary Figure 8. Dual Immunofluorescence of Gh with GnRH1 **(A,B)** or GnRH3 **(C,D)** nerve fibers in the pituitary of female ricefield eels. Gh cells were labeled green with Alexa Flour 488, and GnRH1 and GnRH3 were labeled red with Cy3. The mixture of the rabbit polyclonal antibody AS-691 for GnRH1 (1:7000 dilution) with the mouse anti-Gh (1:500 dilution), or the mouse monoclonal antibody LRH13 for GnRH3 (1:2000 dilution) with the rabbit anti-Gh (1:1000 dilution) were used as the primary antisera. The mixture of Cy3-labeled goat anti-rabbit IgG (H+L) (1:500 dilution) and Alexa Flour 488-labeled goat anti-mouse IgG (H+L) (1:500 dilution), or Cy3-labeled goat anti-mouse IgG (H+L) (1:500 dilution) and Alexa Flour 488-labeled goat anti-rabbit IgG (H+L) (1:500 dilution) were used as the secondary antibodies (Beyotime, Shanghai, China). DAPI was used to stain the nuclei blue, and the neurohypophysis is mostly devoid of stain. The images were captured and overlapped with a Nikon i-E confocal microscope equipped with a CSU-W1 spinning-disk head (Yokogawa, Tokyo, Japan) under the same conditions. **(B)** and **(D)** are higher magnification of the boxed areas in **(A)** and **(C)**, respectively. The overlapping of the red with the green color generated a yellow color (*white arrows*). Sagittal sections of ricefield eel pituitaries were shown here with the rostral (anterior) to the left. The scale bar is 50 μm in **(A)** and **(C)**, and 10 μm in **(B)** and **(D)**.
