# Supplementary material for: A Type IIb, but Not Type IIa, GnRH Receptor Mediates GnRH-Induced Release of Growth Hormone in the Ricefield Eel
Source: Front Endocrinol (Lausanne). 2018 Nov 30;9:721. doi: 10.3389/fendo.2018.00721 (PMC6283897; doi:10.3389/fendo.2018.00721)
Supplement: Supplementary file 12 [file Data_Sheet_10.PDF]

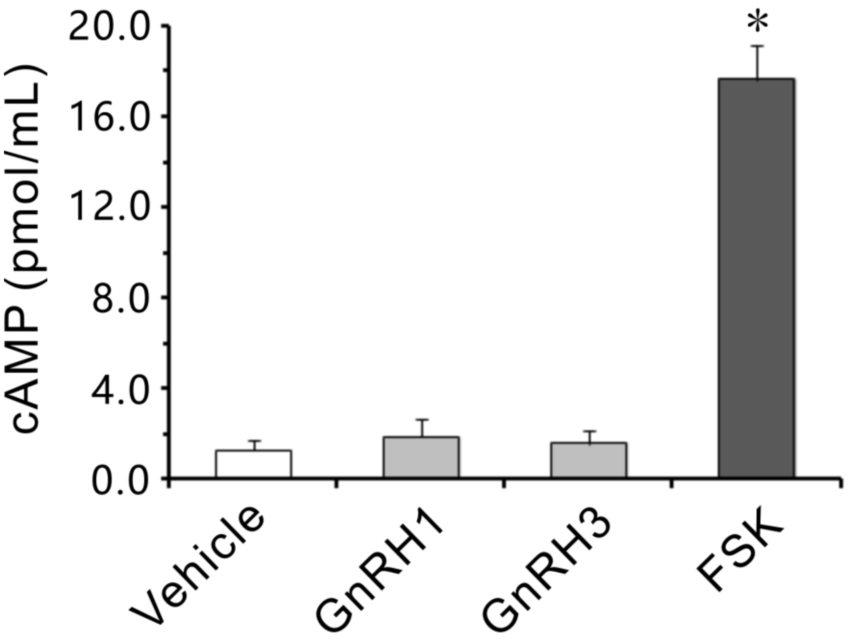


**Supplementary Figure 9.** Effects of 4-h incubation with GnRH1, GnRH3 or forskolin (FSK) on cAMP production in primary pituitary cells of ricefield eels. The cells were pre-incubated for 24 h before being treated with GnRH1 (100 nM), GnRH3 (100 nM), and forskolin (FSK) (10 μM) for 4 h. FSK was used as a positive control. The working solutions of GnRH1, GnRH3, and FSK were diluted from stocks with DMEM, containing 0.1% DMSO. The vehicle control was DMEM with 0.1% DMSO. After drug treatment, the amount of cAMP in the pituitary cells was quantified with a Monoclonal Anti-cAMP Antibody Based Direct cAMP ELISA Kit (80203, NewEast Biosciences, Inc., PA, USA). Results are expressed as measured cAMP concentrations in pituitary cell homogenates (pmol/mL). Bars represent means ± SEM ($n$ = 4). *$P$ < 0.05 vs the vehicle control.
